# Online marketing and brand awareness for HEI: A review and bibliometric analysis

**DOI:** 10.12688/f1000research.127026.1

**Published:** 2023-01-19

**Authors:** Sailaja Bohara, Vashali Bisht, Pradeep Suri, Diksha Panwar, Jyoti Sharma

**Affiliations:** 1 Uttaranchal Institute of Management, Uttaranchal University, Dehradun, Uttarakhand, India; 2 Institute of Professional Studies and Development, Kumaun University, Naintal, India; 3 IILM Graduate School of Management, Noida, Uttar Pradesh, India; 4 DAV Centenary College, Faridabad, Haryana, India

**Keywords:** analysis, Brand awareness, Online marketing, Higher education institutions, Enrollment, Science mapping, Bibliometrix, Technology

## Abstract

**Background:** Many studies have been conducted on higher education institutions (HEI) regarding advertising, promoting, branding, social media marketing, and student enrollment. We investigated the gap in these studies by using bibliometric analysis and comprehensive science mapping in the field of HEI online marketing and brand awareness.

**Methods:** The study used a web-based application, biblioshiny, which comes in the bibliometrix package. The study used the Scopus database to create the data set, given its conventional construction and quality of the sources. The analysis done is descriptive analysis. By using the bibliometrix software, the study showed the authors name, articles, sources, citations, relevant journals and co-citation from the year 2017 to 2022. The time period selected by the study was five years which means that articles published from 2017 to 2022 have been taken for the study.

**Results:** We found that HEI online marketing and brand awareness have not been explored much. The study highlighted that HEI online marketing is a topic that has been developing but has not reached the stage of maturity. Publications on this topic have decreased since 2020. Also, the role of brand awareness in student enrollment decision for HEI requires more investigation. The ways in which brand awareness affects the choice of HEI should be studied. Most of the publications were from sources like higher education, higher education advertising and technology.

**Conclusions:** This subject has been researched, but not much. This paper has given a path for interdisciplinary approaches that can be further explored in the field of higher education and marketing. Further, it gives opportunity to examine publications patterns through different authorships, co-authors, collaborations, relevant sources and citations. The insights of this paper will help education policymakers to devise more creative strategies to increase enrollment. This would give an in-depth understanding of this field to the readers.

## Introduction

Nowadays, higher education has become a very important part of life. People want to join higher education institutions (HEIs) and prepare themselves for a job and a better quality of life ( Brooks *et al.*, 2020). Students are the main customers of HEIs, and therefore their marketing strategies should be devised according to the needs of the students. Proper marketing strategies can affect the enrollment decision of the students ( Farjam and Hongyi, 2015). Technology is imperative to have an upper hand as it guarantees the benefits and endurance of an organization. Before an organization can benefit from any technology, it should become aware of its presence and assess its capacities sufficiently. Technologies are not always created within the HEIs, but are outsourced by other companies; in the era of technology information, they are the key to success, which becomes a challenge for organizations, as they have to consider other external sources for getting the information ( Von, 2005). The means of communication have changed, even within organizations, because of the emergence and rapidly growth of social media ( Aral *et al.*, 2013).

The marketing strategies and policies have changed over time. An organization is represented by the products and services that they are offering, and their success depends on their branding *i.e the brand that they create.* Branding gives the organization the liberty of being recognized by the customers and creates loyal customers. Thus, it increases the probability of being purchased by the customers ( Ukaj, 2016). In the past, branding was only restricted to manufacturing companies and fast-moving consumer goods (FMCG). Now, this scenario has changed. Branding has diversified and has become imperative for HEIs as well. Brand awareness and service quality has to be developed side by side by HEIs to create brand loyalty ( Abbas, 2019). There has been an increase in the student population who wants to attend higher education. This has created an intense competition between the colleges, be it nationally or internationally, for enrollment of the students ( Smith *et al.*, 2017). The population of student who wants to enroll for higher education studies are increasing drastically, because they get a chance to find work in the future from a,professional qualification, which they would get from the institution. The thirst for higher education has no boundaries set for these students, as the selection of a college also depends on their will to relocate themselves to get into the college they want. This factor has also increased the level of competition between these institutions ( Hoxby, 2009 and Susanu and Raducan, 2014).

### Bibliometric analysis

A bibliometric analysis is carried out by analyzing the patterns of published literatures using statistical tools and mathematics ( Singh and Dhir, 2019). It is a method that explores scientific data helping to understand emerging research areas ( Donthu *et al.*, 2021). It is a new concept in the field of business research and therefore, many researchers might not be able to make the full use of bibliometric analysis ( Brown *et al.*, 2020). According to Torraco (2005) it is not easy to arrange a literature review that is very effective in research studies. In the field of research, it is important to understand the gaps of the previous research work to gain access to more knowledge ( Lee and Greenley, 2010). Paré *et al.*, (2015) stated that a literature is effective only when it has made some contribution on the studies that has been conducted previously, and by identifying the areas where further research can be done. Scholars use bibliometric analyses for a variety of reasons, such as to uncover emerging trends in article and journal performance, collaboration patterns, and research constituents, and to explore the intellectual structure of a specific domain in the existing literature.

**Online marketing and HEI**

Demand for education has evolved the education system and therefore, the number of colleges providing education. The competition is intense between these institutions; therefore, online marketing has become very important for these institutions. Further, our study found that online marketing affects the enrollment process of the higher education institution at all the different stages of the process ( Bohara *et al.*, 2022). Observations have shown that social media is pivotal to gain the attention of the students, keeping in touch with them and maintaining the current students. Using social media to communicate with the students may give a good impression about the college to the students ( Omar. T Salem, 2020). According to Ashan (2017), as of late there is competition among colleges for enrolling undergraduates, which has made the colleges to regard students as their customer. The competitions and the demand for high-quality education have made it an important requirement to take on cautious marketing procedures for colleges.

The colleges have to be up to date with the kind of marketing strategies being used on the market by their competitors to look for any new opportunity that can be helpful to face the competition. Higher education today has recognized these promoting blended procedures to confront their opposition ( Jalena Gajic, 2012). Higher education today has recognized these blended promotion strategies to confront their opposition ( Jalena Gajic, 2012). The colleges need to supply the students, stakeholders and anyone who is looking into the college, with rich information as required. Online innovations have offered colleges with devices and techniques that can be utilized to satisfy these requirements ( Alexa *et al.*, 2012). In the present situation, colleges are excited about tracking down new ways and techniques for connecting with prospective students, their graduated class and different partners by utilizing Twitter ( Kowalik, 2011). Internet promoting is an efficient method for publishing data and talk about the benefits that one would acquire from the college (Evans 2009). Marketing has provided more guidance to education institutions. With the utilization of better approaches for advertising strategies, student enlistment has expanded for colleges and has been valuable to see the progressions happening in education sector for colleges ( Uncles, 2018). As per Joana and Maria (2018), colleges have been increasing their investments on internet showcasing. With the increasing utilization of web-based entertainment all over the planet, colleges must be active via online platforms like social media. Being present online doesn't mean simply having a webpage for the college. The college must be dynamic on it. It needs to allow and urge individuals to speak with one another and with the college. One thing that is essential for the colleges is to keep updating their materials and information on their social media site at the right time *i.e* publishing the right post at the right time ( Peruta and Shields, 2018). As per Saichaie and Morphew (2014), where information about college is required, the website becomes imperative as it will give a better knowledge about the college and state the objective of education.

There are many advantages that are offered by online marketing that traditional marketing cannot offer. By online marketing achieving the requirements of the business and the users creating a relationships between the business and its customers and also, fulfilling the requirement of the data ( Wilson and Makau, 2018).

### Brand awareness and HEI

According to Bohara *et al.* (2022) there is an increase in the competition for enrollment between the colleges, which has made brand awareness an essential piece of their advertising movement. The study infers that there is a critical relationship between elements of brand awareness and students' college choice. The article allows to understand the role of brand awareness in each phase of enrollment decision. These organizations ought to look into brand awareness programs more as it might bring about the increment of undergraduates’ enlistment numbers. The review showed that brand awareness is significant for colleges as it influences every phase of student enrollment. Zhang (2020), stated that in the dynamic course of buyers, brand awareness is the basis of marketing activities. High brand awareness has an effect on the customer comprehension process such that they can with less exertion or doubt recollect and separate the brand from others. In the event of low brand awareness, it requires investment to affect the customer discernment process. In this way, buyers favor those organizations who have paid attention to brand to those who have not. Today the advancement of web innovation has changed how organizations and customers communicate through online media; it has united individuals from the entire world ( Chatterjee and Kar, 2020). According to Patil (2017) having a brand name has become imperative in recent years, as people trust in the name of the brands and are able to connect and make decisions only when they are familiar with the name of the brand. Therefore, awareness for the name of the brand is necessary, as it will also make it easy for the customers to make decisions. Therefore, one can say that the amount of awareness that one creates about their brand affects the decision-making process of the consumers. Brand awareness may not just help in taking the decision in the favor of the brand, but also plays a role in creating loyal customers. With the technology shifting towards online platforms, brand awareness by means of using online media has also shown that it affects the decision-making process of the customers. So, brand awareness can be created by means of various online media to increase the probability for the product or the service to be purchased by the customers ( Karam and Saydam, 2015). Brand awareness is also increased by means of online marketing strategies which have the probability of turning the customer in the favor of the brand and loyal ( Hajarian *et al.*, 2021). According to Mirzaei *et al.* (2016) the web page of a college is essential, and those that are active and put good information such as on the services, courses, scholarship information or any grants and other information that are helpful for the viewers, on their pages creates a good impression of their college in front of the students. Nevzat *et al.*, (2016) stated that for colleges and universities, it is useful to use social media as it will help them to build a trustful relationship between them and the name of their college, and the.

Online marketing and brand awareness for higher education has been investigated by the various authors. They identified the research gaps in colleges and brand awareness, colleges and online marketing and college choice of students, but no bibliometric analysis has been done in these literatures. Therefore, this paper presents a bibliometric analysis using descriptive analysis.Bibliometric examination concentrates on utilizing numerical and factual procedures to analyze patterns in published literature reviews ( Singh and Dhir, 2019).

## Methods

**Figure 1.**
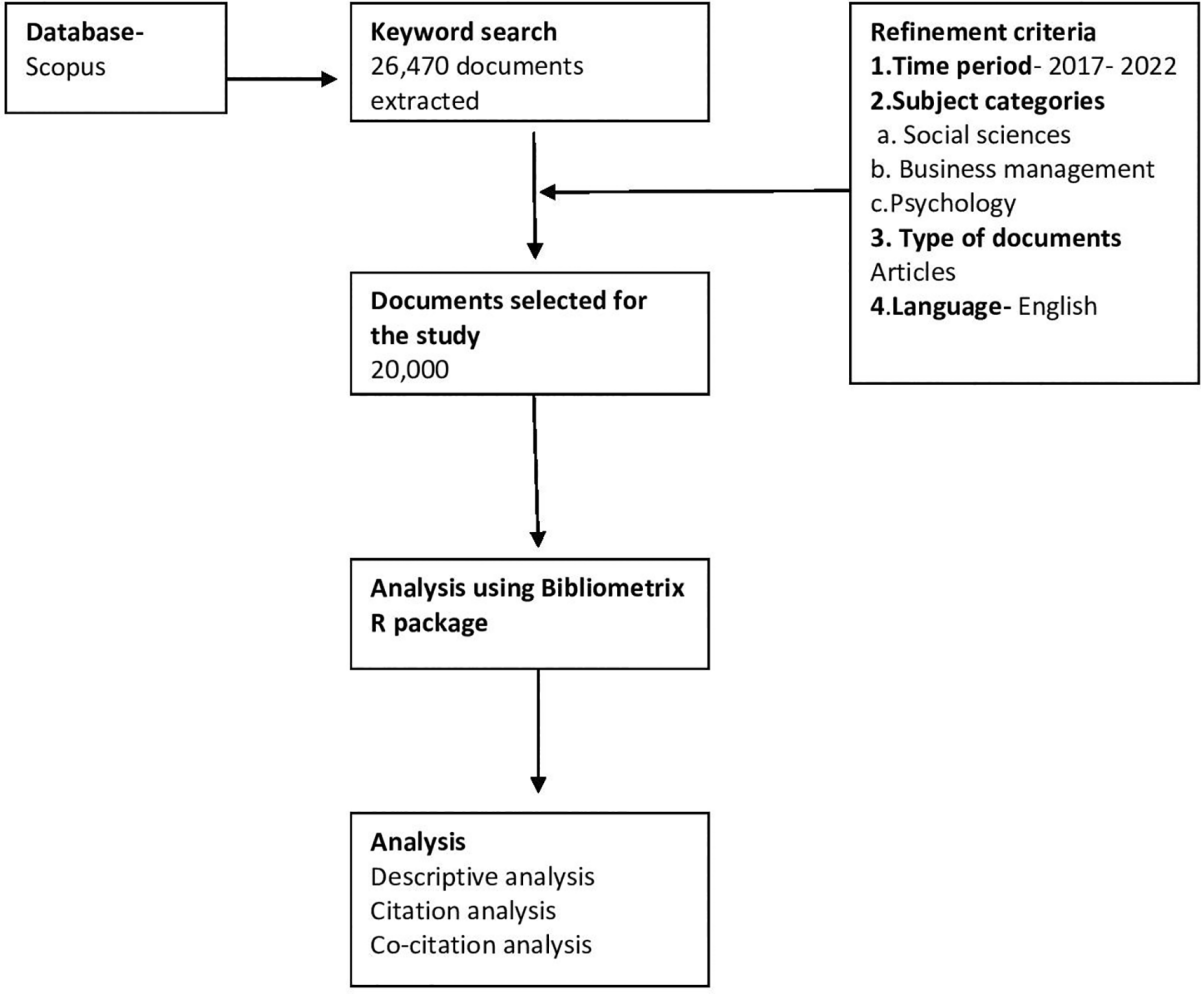
Flowchart for bibliometric analysis.

The data that one uses for bibliometric software are in larger volumes because they come from databases like Web of Science and Scopus. The bibliometric software have raised the interest of researchers in doing bibliometric analysis in the recent years. This analysis through bibliometric software can be done in different business fields and strategies ( Kumar, *et al.*, 2021). Literature reviews aim to assess the literature to recognize potential investigation gaps and highlighting information ( Tranfield *et al.*, 2003). The literature review helps to get the right kind of search keywords, previous literatures and thus, helps in complete analysis through bibliomatrix software ( Saunders *et al.*, 2009). Rowley and Slack (2004) suggested that there should be a structured method for the academic research study, and a design for structuring literatures, way of writing a research study and bibliography. Data needed for this study were extracted after identifying and selecting an appropriate database. For this study, the Scopus database was used and a search for keywords was carried out, as well as a combination of keywords, after which a software tool was used for statistical analyses. Data is used to conduct a descriptive analysis of documents, citations sources and authors ( Ingale and Paluri, 2020). The flowchart for selection of documents for bibliometric analysis can be seen in Figure 1.

### Preparing for data analysis

The study used descriptive analysis. To support the requirement of the software, the data was first downloaded from Scopus database. After this search strategy was used, keywords like ‘higher education institutions’, ‘online marketing strategies’, ‘online brand awareness’, and ‘college enrollment’ were used which extracted 20,000 documents, 4209 sources and types including articles and journals. In order to analyze publication trend, the study time period selected was from the year 2017 to 2022.

*Search strategy for keywords*

There were four keywords that was used in this study, these were, “online marketing strategy”, “online brand awareness”, “college enrollment”, and “higher education institution”. Knowledge of facts on online marketing and brand awareness done by higher education institutions impact on student enrollment were recognize using the search criteria for keywords. These were 1) online marketing of higher education, 2) online brand awareness, 3) online student enrollment 4) brand awareness and student enrollment. The combination was used to get appropriate studies. These combinations were made as they were all related to the topic of the research being conducted.

Refined by- Scopus categories: (Social science, Business management and Psychology).

Document Type: Articles

Language: English

Time period: 2017 to 2022

*Time period*

The trend and knowledge over five years were investigated for this study *i.e.* from 2017 to 2022. Online marketing has recently emerged as the fastest-growing type of marketing strategy, therefore the study focused on this time period.

*Subject category*

Three subjects were used in the refined search category:“Psychology”, “Business management”, and “Social science”. At this stage, 26,470 were extracted.

*Language filter*

After applying the language filter for English, the final documents were extracted by importing the authors, keywords, title and abstracts to biblioshiny and the final number of selected studies was 20,000 documents.

### Selection of bibliometric tool

A bibliometric technique was adopted in this study for a complete science mapping. This technique allows exhaustive bibliometric study that includes data analysis along with visualization. It is not simple to use bibliometric techniques as they require a commercial license to use the tools needed for conducting a bibliomatrix analysis and training to be used. Bibliometrix is an open-source programming which is intended for extensive science planning investigation. It is equipped for constant upgradate and combination with other R statistical packages. Thus, it is generally welcomed by clients (researchers, scholars and academics) and turns out to be profoundly important in the unique field of bibliometric analysis, for descriptive and network analysis. This study dissected the information utilizing Biblioshiny, which is an online application included in Bibliometrix package ( Ingale and Paluri, 2020).

## Results

**Figure 2.**
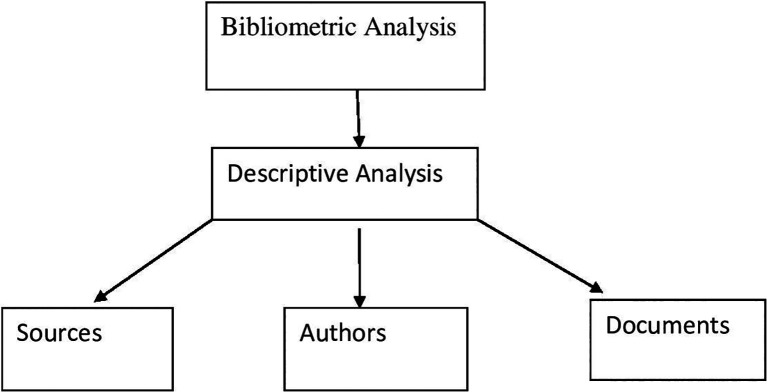
Bibliometric analysis levels.

The study used descriptive analysis which has focused on bibliometric data:Author, sources and documents ( Figure 2).

### Descriptive analysis

*Data set*

Table 1 gives a view of the bibliometric frame; there were 4209 sources which published these documents. The average citation score per document was 4.597 and the collaboration index was 1.74 which indicates that the research was carried out with collaborating with other authors.

**Table 1.** Summary of data set.

Description	Result
Document	20,000
Sources	4,209
Keywords plus (ID)	0
Authors keyword (DE)	0
Period	2017-2022
Average citation per document	4.597
Authors	34,763
Authors appearance	116,451
Authors of single-authored documents	17
Authors of multi-authored documents	34,746
Co-Authors per document	5.82
Documents per author	0.575
Authors per document	1.74
Collaboration index	1.74

*Sources*

**Figure 3.**
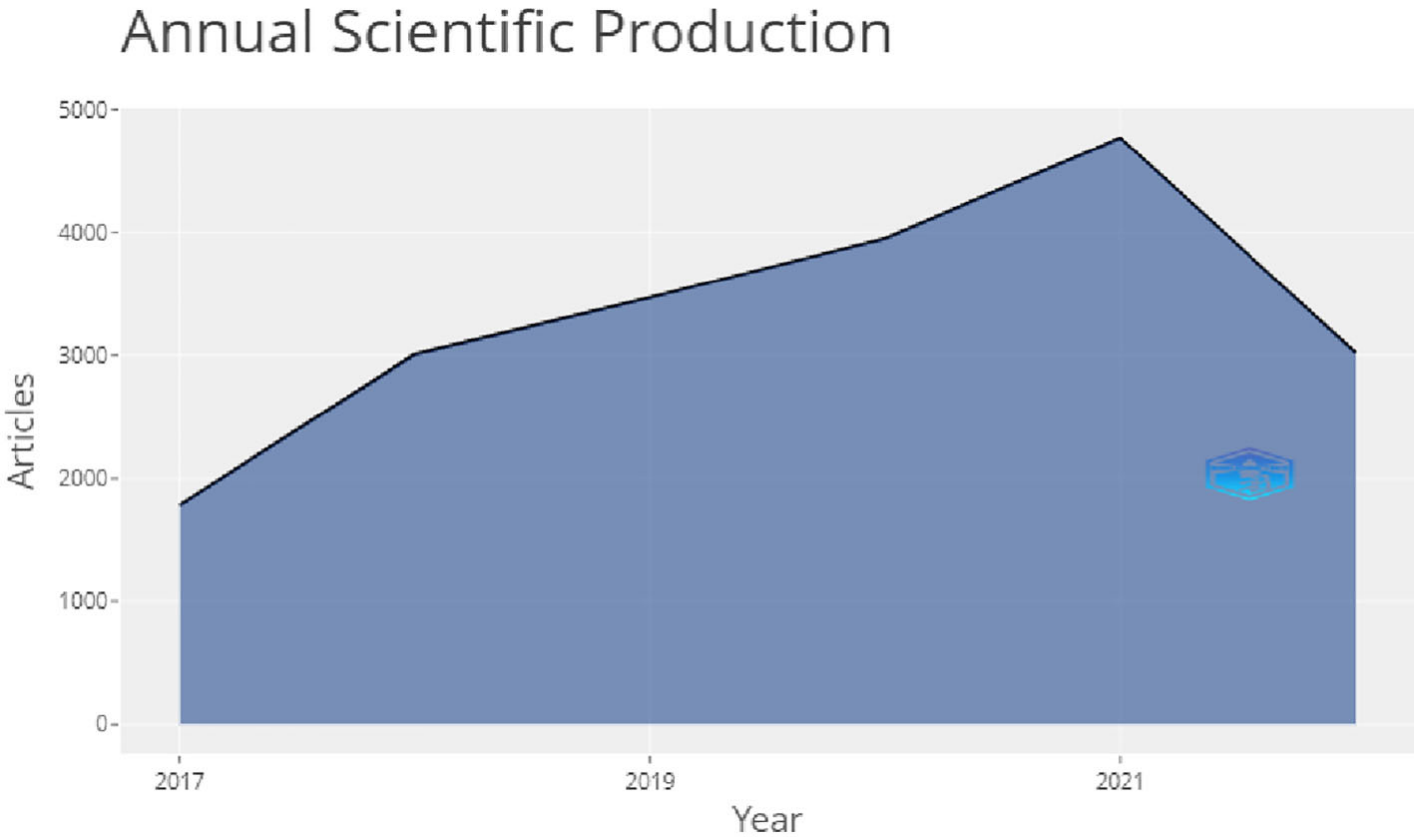
Annual production of articles.

Figure 3 shows the annual scientific production from the year 2017 to 2022.On this figure we can see a sharp surge in the publication volume from 2017 to 2018, as this field of research was gaining importance and was not fully completed *i.e* it did not reached the stage of saturation *.* After that, from 2018 to 2019 the topic was still being researched but not with a sharp surge, as the growth rate decreased. From 2019 to 2020 the research was ongoing but not increasing sharply, with a low production on this field, but after 2020 to 2021 again a sharp surge in the publication volume could be seen which eventually decreased from 2021 to 2022.

In Figure 3, one can see that in 2020 there was a sharp surge, meaning that there were studies being published at this stage, although this was during the coronavirus disease 2019 (COVID-19) pandemic that hit the world in 2020. After, this the graph slopes downwards showing that the production has fallen recently.

**Figure 4.**
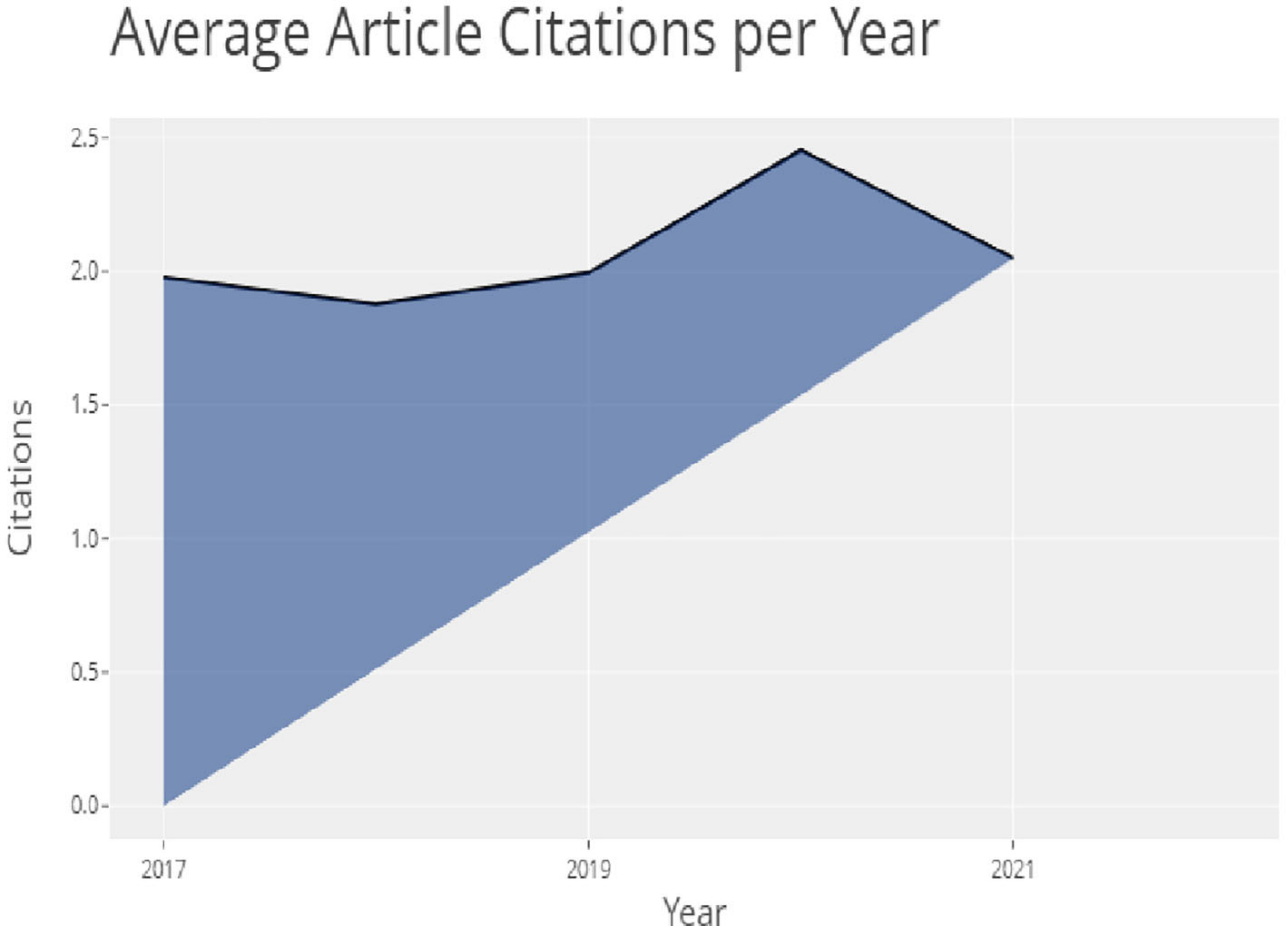
Article citations.

**Figure 5.**
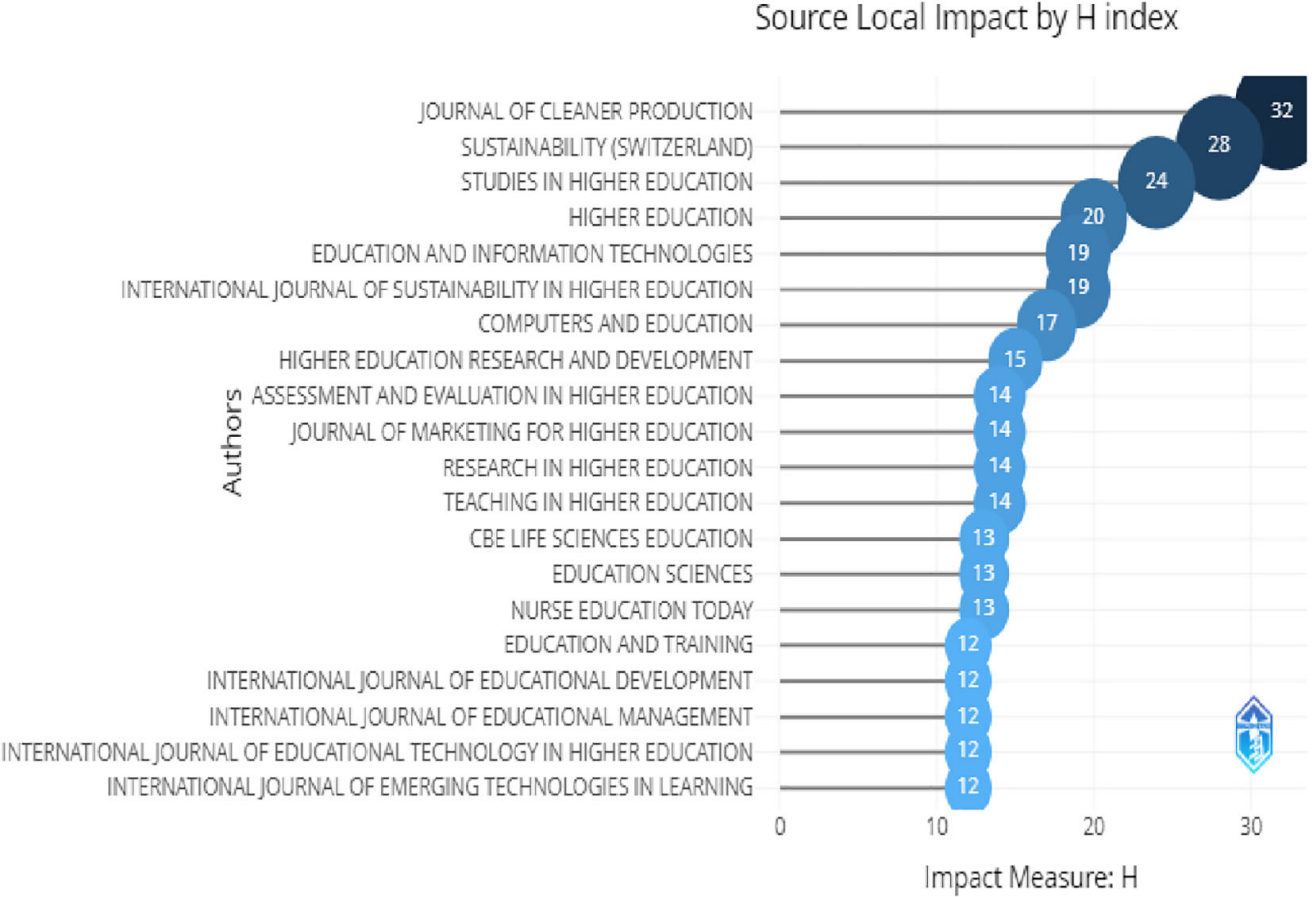
Top 20 impactful sources.

In Figure 4, the top 20 globally most cited documents can be seen. Sustainability, *International Journal of Education, Journal of advertising*, were at the top of the list. In Figure 5, we can see the most relevant sources in which sustainability, studies in higher education, higher education, international journal of sustainability in higher education are at the top of the list. Looking at this information we can say that most of the words used here were related to higher education, higher education and technology, advertising, information and online learning.

**Figure 6.**
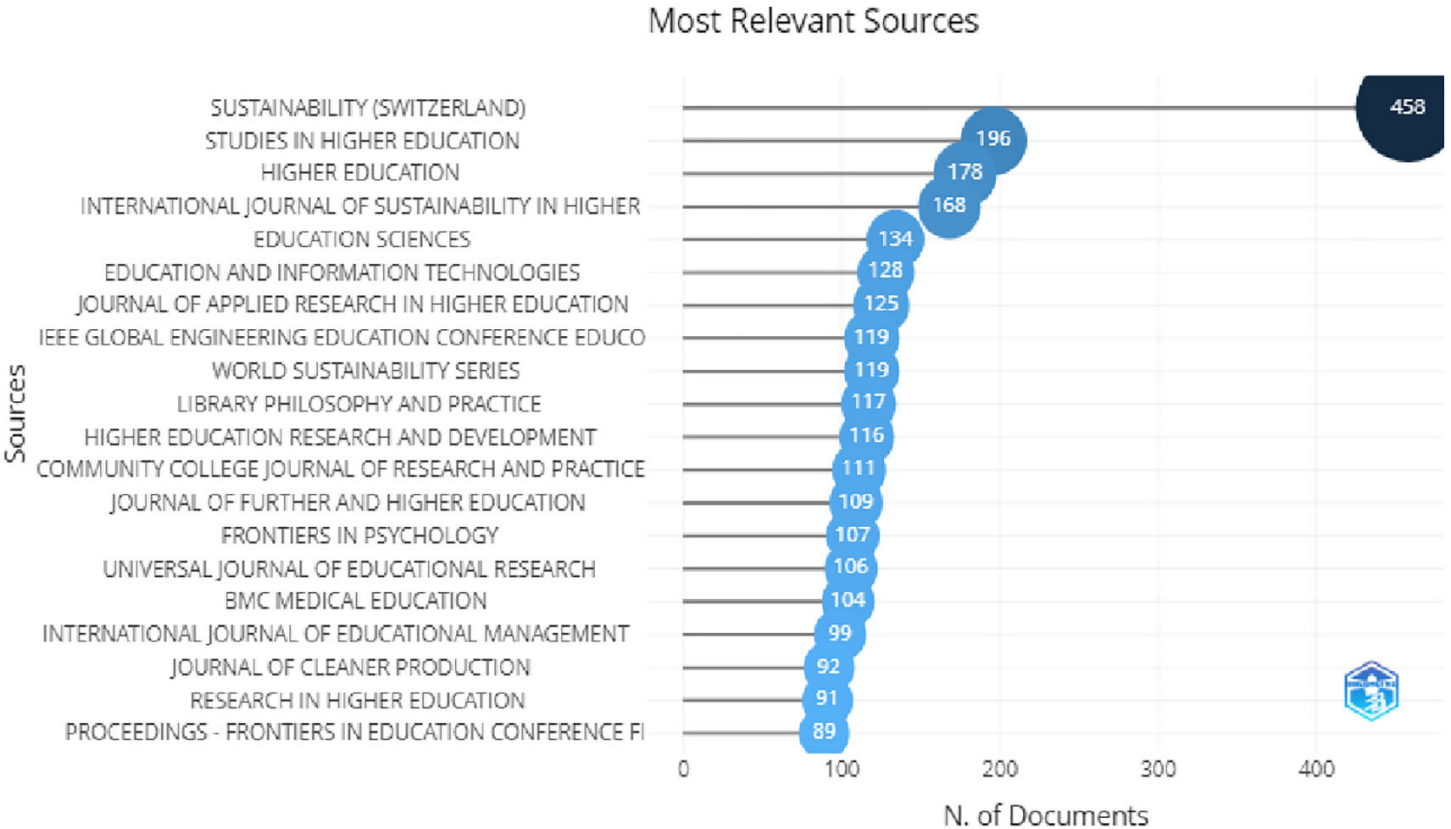
Relevant sources.

Figure 6 shows the effect of these journals. The H-index refers to a most extreme value of "n". Now, "n" refers to the number of journals which have articles published with the least citations. H-index indicates the quality of the journal as well as its impact.

*Authors*

**Figure 7.**
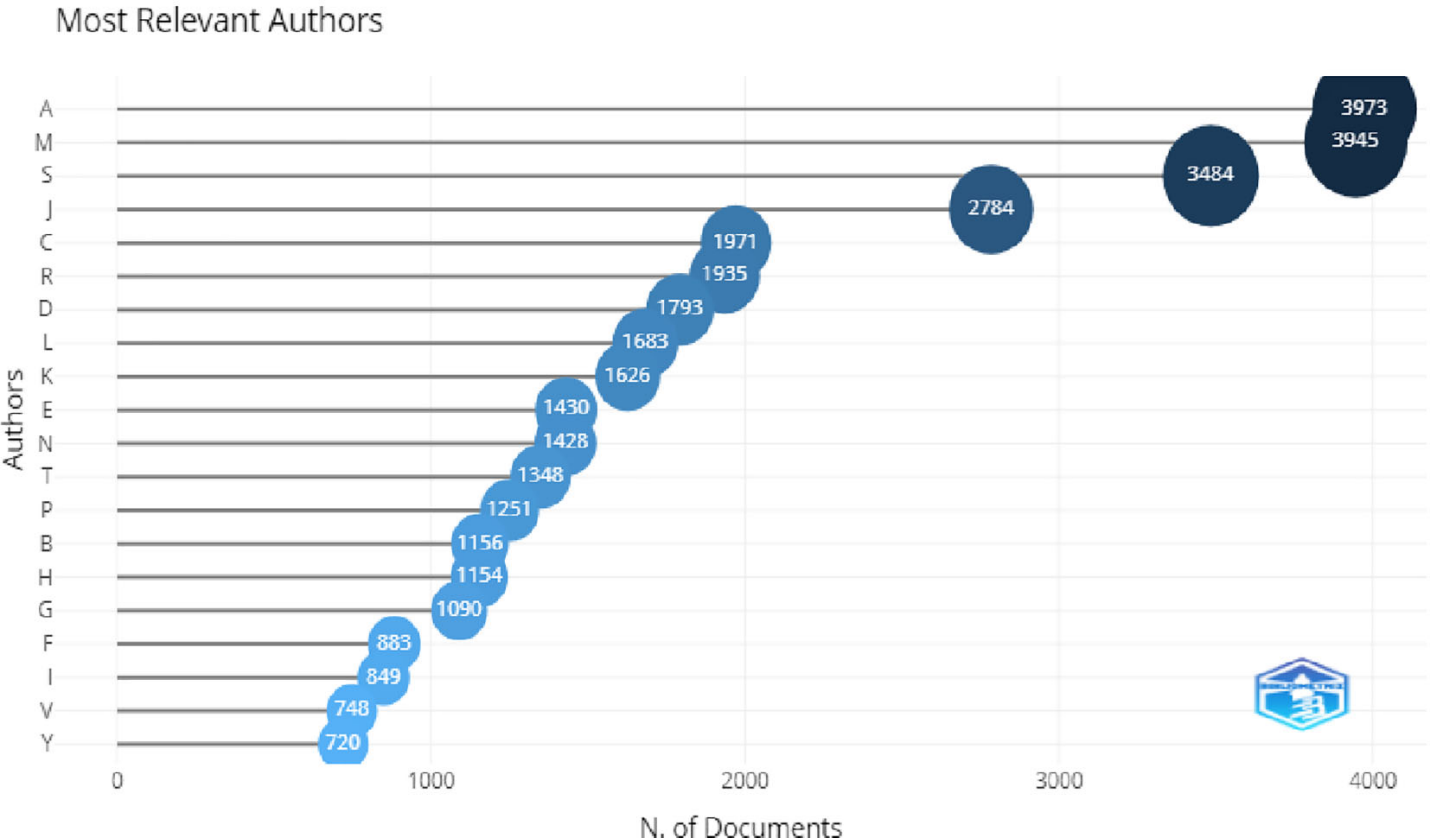
Most relevant authors.

**Figure 8.**
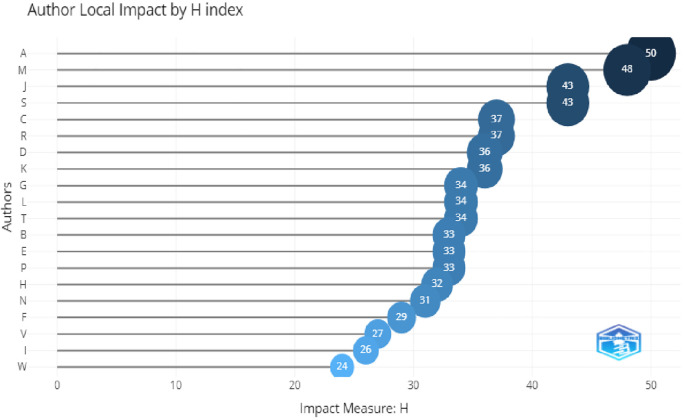
Author impact.

In Figure 7, the top three most relevant authors were A. Aleksander,L. Mishra, and Lou, with the maximum number of publication. The H-index of these authors and citation were also higher, which shows their relevancy. This can be seen in Figure 8.

*Documents*

**Figure 9.**
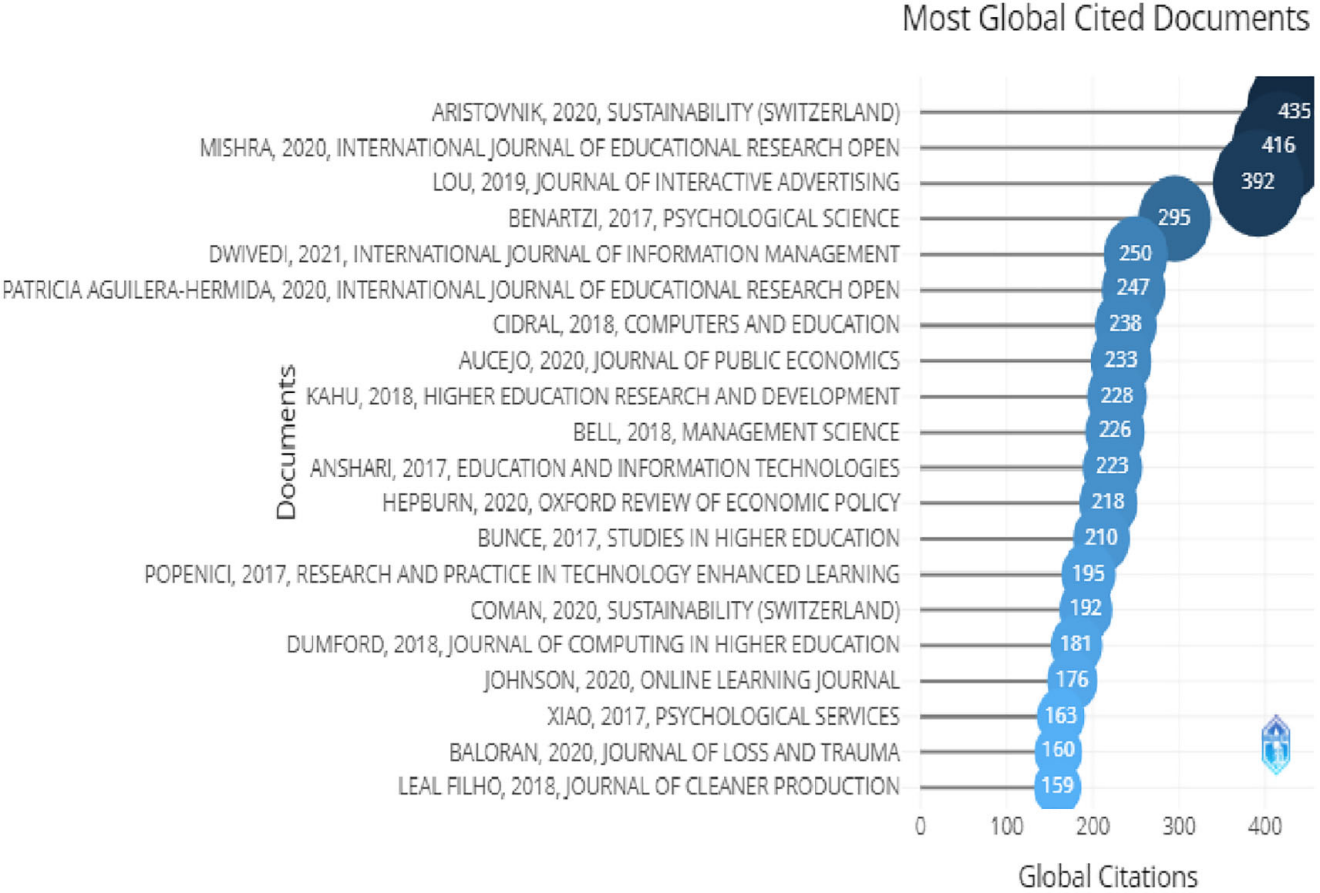
Most globally cited documents.

Figure 9 shows the most cited documents with the author’s name, in this field. There was no article with less than 100 citations and Aristovnik, Mishra and Lou had more than 300 citations. These articles were about advertising, which covered branding and online marketing, higher education and higher education institution.

### Defining the appropriate search terms

The main words that were used were “brand awareness”, “higher education institution”, “online marketing strategies”, “online brand awareness and “college enrollment”. Also, to get a better result, words were combined and used for searching. These combinations were 1) online marketing of higher education, 2) online brand awareness, 3) online student enrollment 4) brand awareness and student enrollment. The combination was used to get the appropriate studies. For example, brand awareness and student enrollment yielded studies related to brand awareness effect on enrollment of students, online marketing showed the various online marketing strategies for online brand awareness and enrollment, and so on. During the search we ensured that all the required work in the study was covered. Subjects included were social sciences, business management and psychology.

## Discussion and conclusions

Paré *et al.* (2015) stated that a publication is effective only when it has made some advancement on the studies that have been conducted previously and also, by identifying the areas where further research can be done. Scholars use bibliometric analysis for a variety of reasons, such as to uncover emerging trends in article and journal performance, collaboration patterns, and research constituents, and to explore the intellectual structure of a specific domain in the existing literature. The results show that from 2017 to 2022 for HEIs, marketing and advertising has become important and they have been using them. The previously published literature was used for analysis to retrieve the important sources, writers and records. The bibliometrix R package, which is a valuable tool for bibliometrics was chosen due to its adaptability and ease of use. The Scopus data base was used for creating the data set, given its conventional construction and quality sources of the journals. According to the data, papers were published on these topics but had not reached the stage of maturity; however,in the year 2020 there was a sharp increase in the publication, which eventually went down after thatyear. In 2020, the world was hit by the COVID-19 pandemic and the educational institutions were closed for some time, but later on the situation had forced the institutions to use digital platform. The COVID-19 pandemic caused countless setbacks around the world, with significant social and financial effects. UNESCO reported that around one billion students had been compelled to remain at home because of the crisis, and the scale and speed with which schools and colleges had been shut was overwhelming ( Giancola and Piromalli, 2020). To face this crisis, even the schools and colleges could not stop from transferring their activities into digital platforms ( Turcanu *et al.*, 2020). The majority of the publications was from sources like higher education, higher education advertising and technology. This paper, therefore, has given a path for interdisciplinary approaches that can be further explored in the field of higher education and marketing. Further, this paper was an opportunity to examine the pattern of publications by investigating the different authorship, co-authors, collaborations, relevant sources and citations. The insights of this paper will help education policymakers to devise more creative strategies to increase enrollment. This would give an in-depth understanding of this field. Further, for academic researchers, this paper will open many ways in which one could explore and contribute in the educational industry by diversifying the research in areas, where the publications have not reached their maturity level. A combination of keywords was used in this paper: 1) online marketing of higher education, 2) online brand awareness, 3) online student enrollment 4) brand awareness and student enrollment. The bibliometric analysis showed that higher education and marketing are topics which have been explored, resulting in related papers being published, but effects of online marketing of higher education on students’ enrollment and brand awareness role in higher education and its importance are areas which have not been explored. So, our study gives a path for further research.

### Direction for future research

The study used a bibliometric analysis, but only descriptive analysis has been done. So, this study can be examined further by using network analysis. Further, the database used for this study was Scopus, although there are other good databases that also can be used like Web of Science. From this study, we can see that there are not many publications that involve higher education, online marketing and brand awareness and student enrollment all together, while nowadays these are the strategies that help the colleges face competition. There is a need to explore the contribution from scholars and practitioners in this field for getting deeper theoretical and practical insights.

## Data Availability

Figshare: Online Marketing And Brand Awareness For HEI: A Review And Bibliometric Analysis, https://doi.org/10.6084/m9.figshare.21276603 ( Bohara *et al.*, 2022) This project contains the following underlying data:
• Data file 1. (CSV. File of the bibliometric analysis done using Scopus database) Data are available under the terms of the Creative Commons Zero “No rights reserved” data waiver (CC0 1.0 Public domain dedication).
