# Online marketing and brand awareness for HEI: A review and bibliometric analysis

**DOI:** 10.12688/f1000research.127026.2

**Published:** 2024-05-13

**Authors:** Sailaja Bohara, Vashali Bisht, Pradeep Suri, Diksha Panwar, Jyoti Sharma

**Affiliations:** 1 Uttaranchal Institute of Management, Uttaranchal University, Dehradun, Uttarakhand, India; 2 Institute of Professional Studies and Development, Kumaun University, Naintal, India; 3 IILM Graduate School of Management, Noida, Uttar Pradesh, India; 4 DAV Centenary College, Faridabad, Haryana, India

**Keywords:** analysis, Brand awareness, Online marketing, Higher education institutions, Enrollment, Science mapping, Bibliometrix, Technology

## Abstract

**Background:**

This study conducted a comprehensive bibliometric analysis to identify the gaps in the existing literature related to Online marketing and brand awareness strategies for HEI. It has evaluated the current state of the literature on the given topic showing the pivotal role of online marketing and brand awareness in higher education for enrollment.

**Methods:**

The study used a web-based application, Biblioshiny, which comes in the bibliometrix package. The study used the Scopus database to create the data set, given its conventional construction and quality of the sources. The analysis done is descriptive. By using the bibliometrix software, the study showed the authors name, articles, sources, citations, relevant journals and co-citation from the year 2017 to 2022. The time period selected by the study was five years which means that articles published from 2017 to 2022 have been taken for the study.

**Results:**

We found that HEI online marketing and brand awareness have not been explored much as it has not reached the stage of maturity. Most of the publication was done during the time of Covid-19. Also, the role of brand awareness in student enrollment decisions for HEI requires more investigation. Top most publications their sources and top authors are identified.

**Conclusions:**

Bibliometric analysis has provided valuable insights into the seminal work, emerging trends, and the gap in the study. This area of study has been explored but not as much as challenges, and the effectiveness of online marketing tools like seo, sem,ppc and more has not been measured. Further, this paper allows researchers to study by examining the pattern of publications by seeing the different authorships, co-authors, collaborations, relevant sources, and citations. The insights of this paper will help education policymakers devise more creative strategies to increase enrollment, ensuring sustained relevance and competitive advantage in higher education institutions .

## Introduction

Nowadays, higher education has become a very important part of life. People want to join higher education institutions (HEIs) and prepare themselves for a jobs and a better quality of life ( Brooks *et al.*, 2020). Students are the main customers of HEIs, and therefore their marketing strategies should be devised according to the needs of the students. Proper marketing strategies can affect the enrollment decision of the students ( Farjam and Hongyi, 2015). Technology is imperative to have an upper hand as it guarantees the benefits and endurance of an organization. Before an organization can benefit from any technology, it should become aware of its presence and assess its capacities sufficiently. Technologies are not always created within the HEIs, but are outsourced by other companies; in the era of technology information, they are the key to success, which becomes a challenge for organizations, as they have to consider other external sources for getting the information ( Von, 2005). The means of communication have changed, even within organizations, because of the emergence and rapid growth of social media ( Aral *et al.*, 2013).

The marketing strategies and policies have changed over time. An organization is represented by the products and services that they are offering, and their success depends on their branding *i.e. the brand that they create.* Branding gives the organization the liberty of being recognised by the customers and creates loyal customers. Thus, it increases the probability of being purchased by the customers ( Ukaj, 2016). In the past, branding was only restricted to manufacturing companies and fast-moving consumer goods (FMCG). Now, this scenario has changed. Branding has diversified and has become imperative for HEIs as well. Brand awareness and service quality must be developed side by side by HEIs to create brand loyalty ( Abbas, 2019). There has been an increase in the student population who want to attend higher education. This has created an intense competition between the colleges, be it nationally or internationally, for enrollment of the students ( Smith *et al.*, 2017). The population of students who wants to enroll for higher education studies are increasing drastically, because they get a chance to find work in the future from a, professional qualification, which they would get from the institution.

The thirst for higher education has no boundaries set for these students, as the selection of a college also depends on their will to relocate themselves to get into the college they want. This factor has also increased the level of competition between these institutions ( Hoxby, 2009; Susanu and Raducan, 2014). Therefore, this study by analysing the patterns of published literature by using statistical tools and, mathematics bibliometric analysis is done ( Singh and Dhir, 2019). It is a method that explores scientific data helping to understand the areas that are emerging ( Donthu *et al.*, 2021). It is a new concept in the field of business research and so, it happens that many researchers are not able to make full use of bibliometric analysis ( Brown *et al.*, 2020). According to Torraco (2005) it is not easy to arrange a literature review that is very effective in research studies. In the field of research, it is important to understand the gaps in the previous research work and work on it to gain access to more knowledge ( Lee, 2009). Pare *et al.* (2015) stated that literature is effective only when it has made some contribution to the studies that have been conducted previously and by identifying the areas where more and further research can be done. Scholars use bibliometric analysis for a variety of reasons, such as to uncover emerging trends in article and journal performance, collaboration patterns, and research constituents, and to explore the intellectual structure of a specific domain in the existing literature.

Therefore, the present study has provided an overview of the existing research work on the related topic and has highlighted the outputs:

1. Top publication work
2. The top authors
3. Emerging trends
4. Future direction

**Online marketing and HEI**

Demand for education has evolved the education system and therefore, the number of colleges providing education. The competition is intense between these institutions; therefore, online marketing has become very important for these institutions. Further, our study found that online marketing affects the enrollment process of higher education institutions at all the different stages of the process ( Bohara *et al.*, 2022). Observations have shown that social media is pivotal to gaining the attention of the students, keeping in touch with them and maintaining the current students. Using social media to communicate with the students may give a good impression about the college to the students ( Omar. T Salem, 2020). According to Ashan (2017), as of late, there is competition among colleges for enrolling undergraduates, which has made the colleges regard students as their customer. The competition and the demand for high-quality education have made it an important requirement to take on cautious marketing procedures for colleges.

The colleges must be up to date with the kind of marketing strategies being used on the market by their competitors to look for any new opportunity that can be helpful to face the competition. Higher education today has recognized these promoting blended procedures to confront their opposition ( Jalena Gajic, 2012). Higher education today has recognized these blended promotion strategies to confront their opposition ( Jalena Gajic, 2012). The colleges need to supply the students, stakeholders and anyone who is looking into the college, with rich information as required. Online innovations have offered colleges with devices and techniques that can be utilized to satisfy these requirements ( Alexa *et al.*, 2012). In the present situation, colleges are excited about tracking down new ways and techniques for connecting with prospective students, their graduated class, and different partners by utilizing Twitter ( Kowalik, 2011). Internet promotion is an efficient method for publishing data and talking about the benefits that one would acquire from the college (Evans 2009). Marketing has provided more guidance to educational institutions. With the utilization of better approaches for advertising strategies, student enlistment has expanded for colleges and has been valuable to see the progressions happening in the education sector for colleges ( Uncles, 2018). As per Joana and Maria (2018), colleges have been increasing their investments in internet showcasing. With the increasing utilization of web-based entertainment all over the planet, colleges must be active via online platforms like social media. Being present online doesn’t mean simply having a webpage for the college. The college must be dynamic in it. It needs to allow and urge individuals to speak with one another and with the college. One thing that is essential for the colleges is to keep updating their materials and information on their social media site at the right time *i.e* publishing the right post at the right time ( Peruta and Shields, 2018). As per Saichaie and Morphew (2014), where information about college is required, the website becomes imperative as it will give better knowledge about the college and state the objective of education.

Many advantages are offered by online marketing that traditional marketing cannot offer. Online marketing achieves the requirements of the business and the users creating relationships between the business and its customers and also, fulfilling the requirement of the data ( Wilson and Makau, 2018).

### Brand awareness and HEI

According to Bohara *et al.* (2022) there is an increase in the competition for enrollment between the colleges, which has made brand awareness an essential piece of their advertising movement. The study infers that there is a critical relationship between elements of brand awareness and students’ college choices. The article allows us to understand the role of brand awareness in each phase of the enrollment decision. These organizations ought to investigate brand awareness programs more as it might bring about an increment of undergraduates’ enlistment numbers. The review showed that brand awareness is significant for colleges as it influences every phase of student enrollment. Zhang (2020), stated that in the dynamic course of buyers, brand awareness is the basis of marketing activities. High brand awareness influences the customer comprehension process such that they can with less exertion or doubt recollect and separate the brand from others. In the event of low brand awareness, it requires investment to affect the customer discernment process. In this way, buyers favor those organizations who have paid attention to the brand to those that have not. Today the advancement of web innovation has changed how organizations and customers communicate through online media; it has united individuals from the entire world ( Chatterjee and Kar, 2020). According to Patil (2017) having a brand name has become imperative in recent years, as people trust in the name of the brands and can connect and make decisions only when they are familiar with the name of the brand. Therefore, awareness of the name of the brand is necessary, as it will also make it easy for the customers to make decisions. Therefore, one can say that the amount of awareness that one creates about their brand affects the decision-making process of the consumers. Brand awareness may not just help in deciding in the favor of the brand, but also plays a role in creating loyal customers. With technology shifting towards online platforms, brand awareness using online media has also shown that it affects the decision-making process of the customers. So, brand awareness can be created utilizing various online media to increase the probability for the product or the service to be purchased by the customers ( Karam and Saydam, 2015). Brand awareness is also increased utilizing online marketing strategies which have the probability of turning the customer in the favor of the brand and loyal ( Hajarian *et al.*, 2021). According to Mirzaei *et al.* (2016) the web page of a college is essential, and those that are active and put good information such as on the services, courses, scholarship information or any grants and other information that are helpful for the viewers, on their pages creates a good impression of their college in front of the students. Nevzat *et al.*, (2016) stated that for colleges and universities, it is useful to use social media as it will help them to build a trustful relationship between them and the name of their college, and the.

Online marketing and brand awareness for higher education has been investigated by various authors. They identified the research gaps in colleges and brand awareness, colleges and online marketing and college choice of students, but no bibliometric analysis has been done in these literatures. Therefore, this paper presents a bibliometric analysis using descriptive analysis. Bibliometric examination concentrates on utilizing numerical and factual procedures to analyze patterns in published literature reviews ( Singh and Dhir, 2019).

## Methods

**Figure 1.**
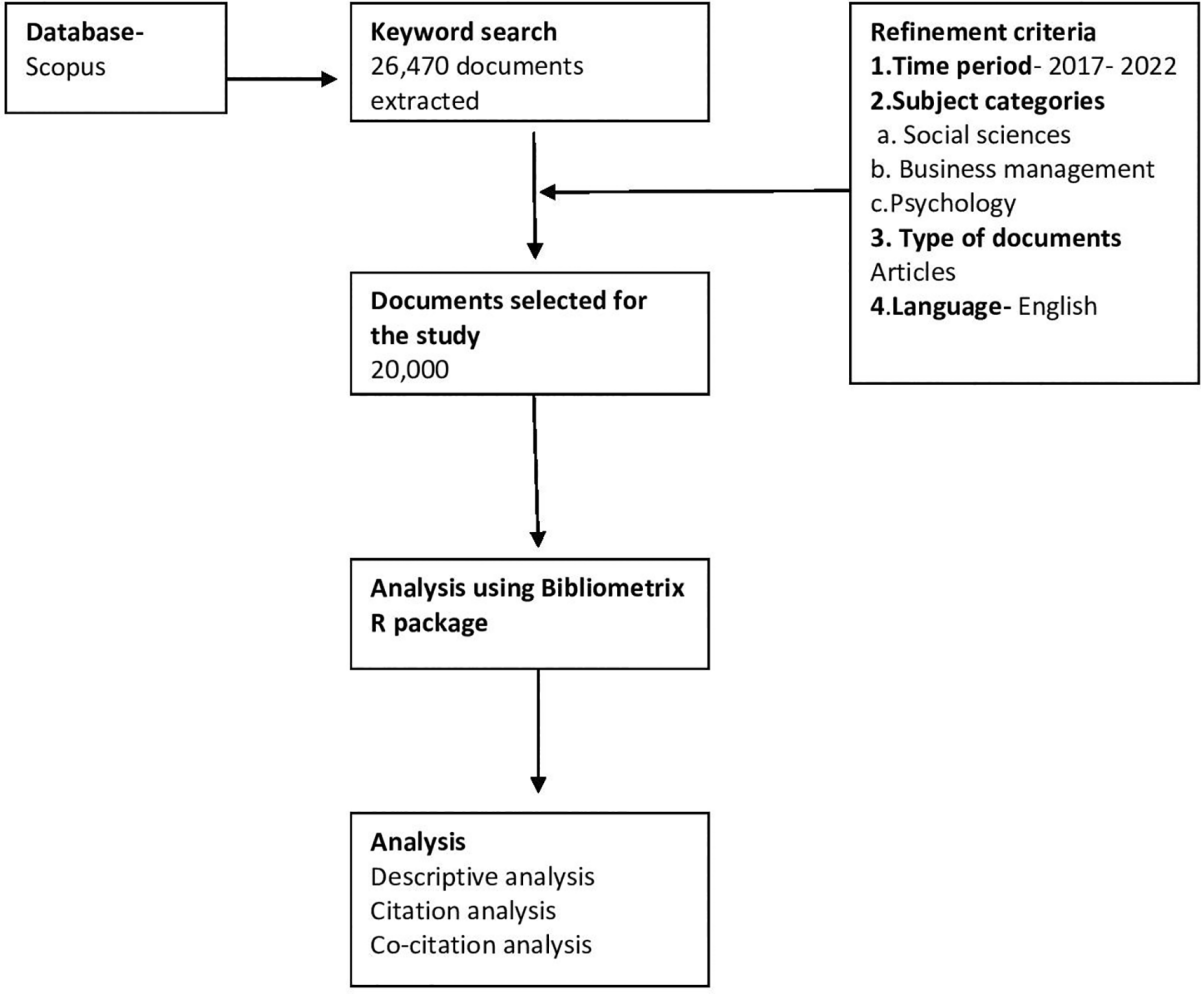
Flowchart for bibliometric analysis.

The study used bibliometric analysis. The data that one uses for bibliometric software is in larger volumes because they come from databases like Web of Science and Scopus. Bibliometric software has raised the interest of researchers in doing bibliometric analysis in recent years. This analysis through bibliometric software can be done in different business fields and strategies ( Kumar, *et al.*, 2021). Literature reviews aim to assess the literature to recognize potential investigation gaps and highlight information ( Tranfield *et al.*, 2003). The literature review helps to get the right kind of search keywords, and previous literature and thus, helps in complete analysis through bibliometrics software ( Saunders *et al.*, 2009). Rowley and Slack (2004) suggested that there should be a structured method for academic research study, and a design for structuring literature, a way of writing a research study and a bibliography. Data needed for this study were extracted after identifying and selecting an appropriate database. For this study, the Scopus database was used and a search for keywords was carried out, as well as a combination of keywords, after which a software tool was used for statistical analyses. Data is used to conduct a descriptive analysis of documents, citations sources and authors ( Ingale and Paluri, 2020). The flowchart for the selection of documents for bibliometric analysis can be seen in Figure 1.

### Preparing for data analysis

The study used descriptive analysis. To support the requirement of the software, the data was first downloaded from Scopus database (data type.CSV). After this search strategy was used, keywords like ‘higher education institutions’, ‘online marketing strategies’, ‘online brand awareness’, and ‘college enrollment’ were used which extracted 20,000 documents, 4209 sources and types including articles and journals. To analyze publication trends, the study period selected was from the year 2017 to 2022.

*Search strategy for keywords*

Four keywords used in this study, these were, “online marketing strategy” and “online brand awareness” and “college enrollment”, and “higher education institution”.

Knowledge of facts on online marketing and brand awareness done by higher education institutions impact on student enrollment were recognized using the search criteria for keywords. These were 1) online marketing of higher education, 2) online brand awareness, 3) online student enrollment 4) brand awareness and student enrollment. The combination was used to get appropriate studies. These combinations were made as they were all related to the topic of the research being conducted.

Refined by- Scopus categories: (Social science, Business Management and Psychology).

Document Type: Articles

Language: English

Time period: 2017 to 2022

*Time period*

The trend and knowledge over six years were investigated for this study *i.e.* from 2017 to 2022. Online marketing therefore recently emerged as the fastest-growing type of marketing strategy, therefore the study focused on this period.

*Subject category*

Three subjects were used in the refined search category: “Psychology”, “Business management”, and “Social science”. At this stage, 26,470 were extracted.

*Language filter*

After applying the language filter for English, the final documents were extracted by importing the authors, keywords, titles and abstracts to biblioshiny and the final number of selected studies was 20,000 documents.

### Selection of bibliometric tool

A bibliometric technique was adopted in this study for a complete science mapping. This technique allows exhaustive bibliometric study that includes data analysis along with visualization. It is not simple to use bibliometric techniques as they require a commercial license to use the tools needed for conducting a bibliomatrix analysis and training to be used. Bibliometrix is an open-source program which is intended for extensive science planning investigation. It is equipped for constant upgradation and combination with other R statistical packages. Thus, it is generally welcomed by clients (researchers, scholars, and academics) and turns out to be profoundly important in the unique field of bibliometric analysis, for descriptive and network analysis. This study dissected the information utilizing Biblioshiny, which is an online application included in the Bibliometrix package ( Ingale and Paluri, 2020).

## Results

**Figure 2.**
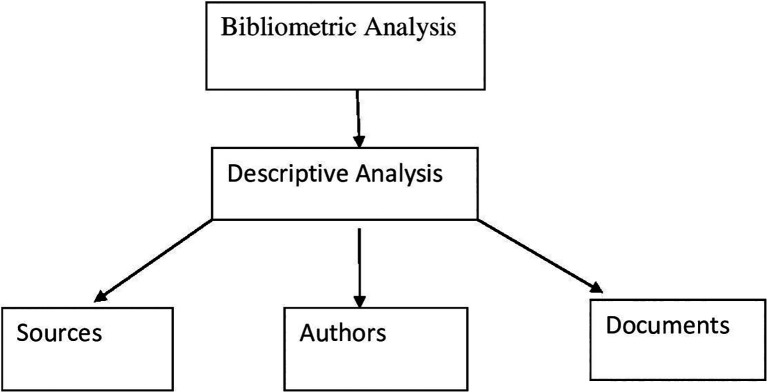
Bibliometric analysis levels.

The study used descriptive analysis which has focused on bibliometric data: Author, sources, and documents ( Figure 2).

### Descriptive analysis

*Data set*

Table 1 gives a view of the bibliometric frame; there were 4209 sources which published these documents. The average citation score per document was 4.597 and the collaboration index was 1.74 which indicates that the research was carried out by collaborating with other authors.

**Table 1.** Summary of data set.

Description	Result
Document	20,000
Sources	4,209
Keywords plus (ID)	0
Authors keyword (DE)	0
Period	2017-2022
Average citation per document	4.597
Authors	34,763
Authors appearance	116,451
Authors of single-authored documents	17
Authors of multi-authored documents	34,746
Co-Authors per document	5.82
Documents per author	0.575
Authors per document	1.74
Collaboration index	1.74

*Sources*

**Figure 3.**
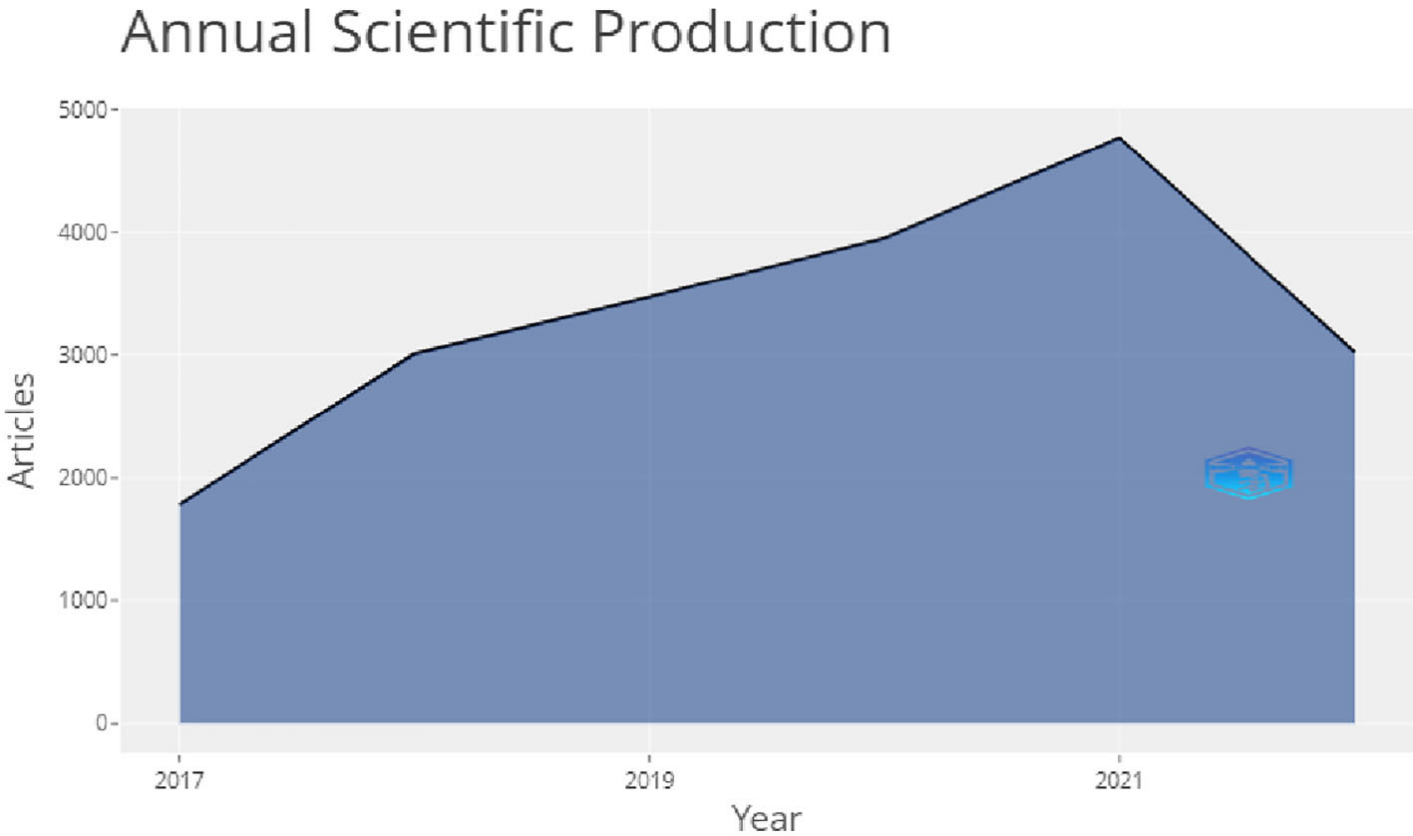
Annual production of articles.

Figure 3 shows the annual scientific production from the year 2017 to 2022.On this figure we can see a sharp surge in the publication volume from 2017 to 2018, as this field of research was gaining importance and was not fully completed *i.e.* it did not reach the stage of saturation. After that, from 2018 to 2019 the topic was still being researched but not with a sharp surge, as the growth rate decreased. From 2019 to 2020 the research was ongoing but not increase sharply, with a low production on this field, but after 2020 to 2021 again a sharp surge in the publication volume could be seen which eventually decreased from 2021 to 2022.

In Figure 3, one can see that in 2020 there was a sharp surge, meaning that studies were being published at this stage, although this was during the coronavirus disease 2019 (COVID-19) pandemic that hit the world in 2020. After this, the graph slopes downwards showing that production has fallen recently.

**Figure 4.**
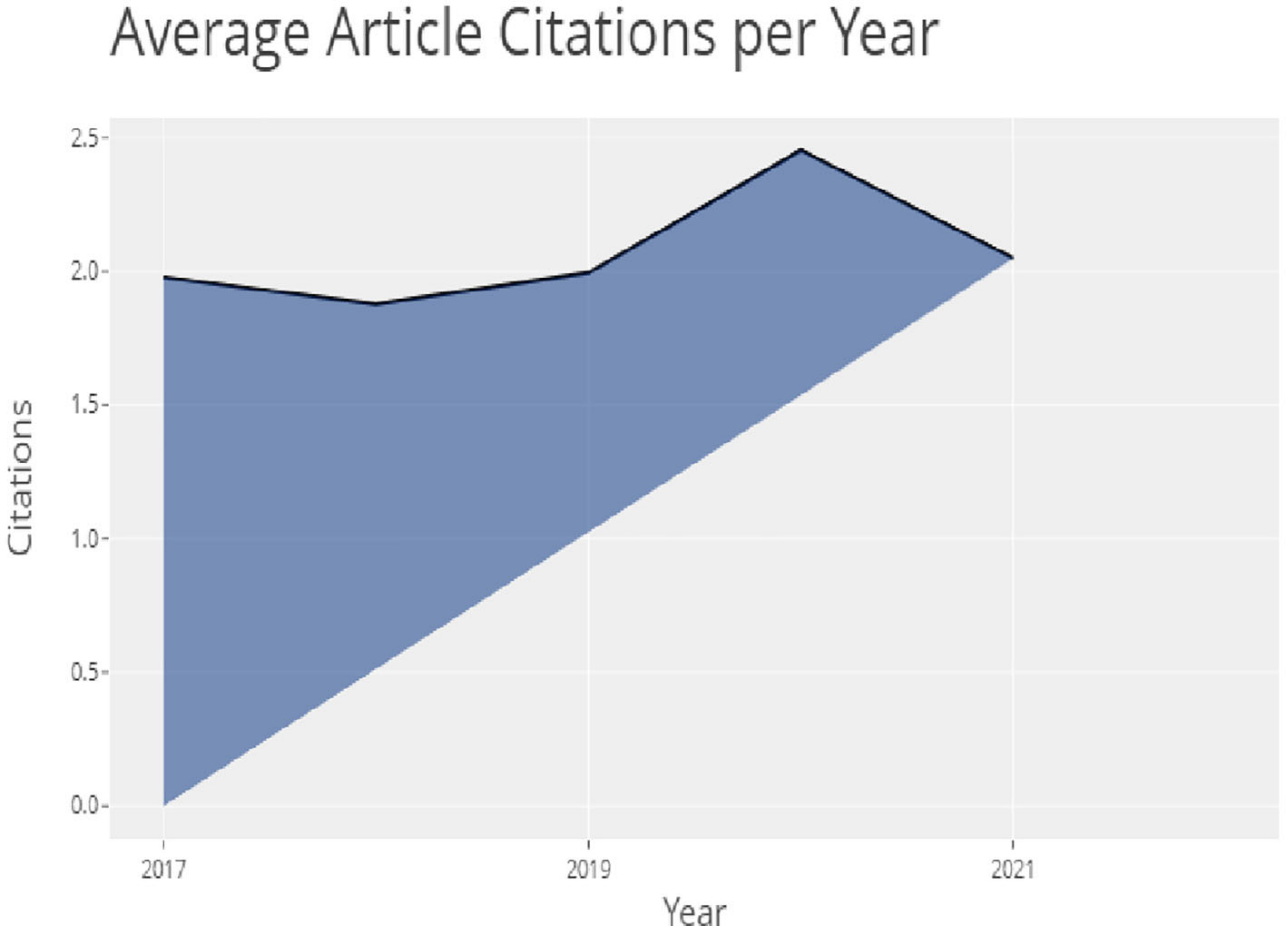
Article citations.

The above Table 2 shows the top 10 globally cited documents and their citations and in Figure 4, the top 20 globally most cited documents can be seen. In which “Impacts Of The Covid-19 Pandemic On Life Of Higher Education Students: A Global Perspective” ( Aristovnik *et al.*, 2020) has been cited the most followed by “Online Teaching-Learning In Higher Education During Lockdown Period Of Covid-19 Pandemic” ( Mishra *et al.*, 2020) showing that covid-19 was the most important reason for higher education institutions all around the world for adopting online marketing strategies and technologies.

**Table 2.** Top 10 globally cited documents.

Author	Source	Title	Total citation	Total citation per Year
Aristovnik, 2020	“International Journal Of Sustainability In Higher Education”	“Impacts Of The Covid-19 Pandemic On Life Of Higher Education Students: A Global Perspective”	435	145
Mishra, 2020	“International Journal Of Educational Research Open”	“Online Teaching-Learning In Higher Education During Lockdown Period Of Covid-19 Pandemic”	416	138.667
Lou, 2019	“Journal Of Interactive Advertising”	“Influencer Marketing: How Message Value And Credibility Affect Consumer Trust Of Branded Content On Social Media”	392	98
Benartzi, 2017	“Psychological Science”	“Should Governments Invest More In Nudging?”	295	98
Dwivedi, 2020	“International Journal Of Information Management”t	“Setting The Future Of Digital And Social Media Marketing Research: Perspectives And Research Propositions”	250	125
Patricia Aguilera-Hermida, 2020	“International Journal Of Educational Research Open”	“College Students’ Use And Acceptance Of Emergency Online Learning Due To Covid-19”	247	82.333
Cidral, 2018	“Computers & Education”	“The Effects Of A Digital Formative Assessment Tool On Mathematics Achievement And Student Motivation: Results Of A Randomized Experiment”	238	47.6
Aucejo, 2020	“Journal Of Public Economics”	“The Impact of Covid-19 On Student Experiences And Expectations: Evidence From A Survey”	233	77.667

**Figure 5.**
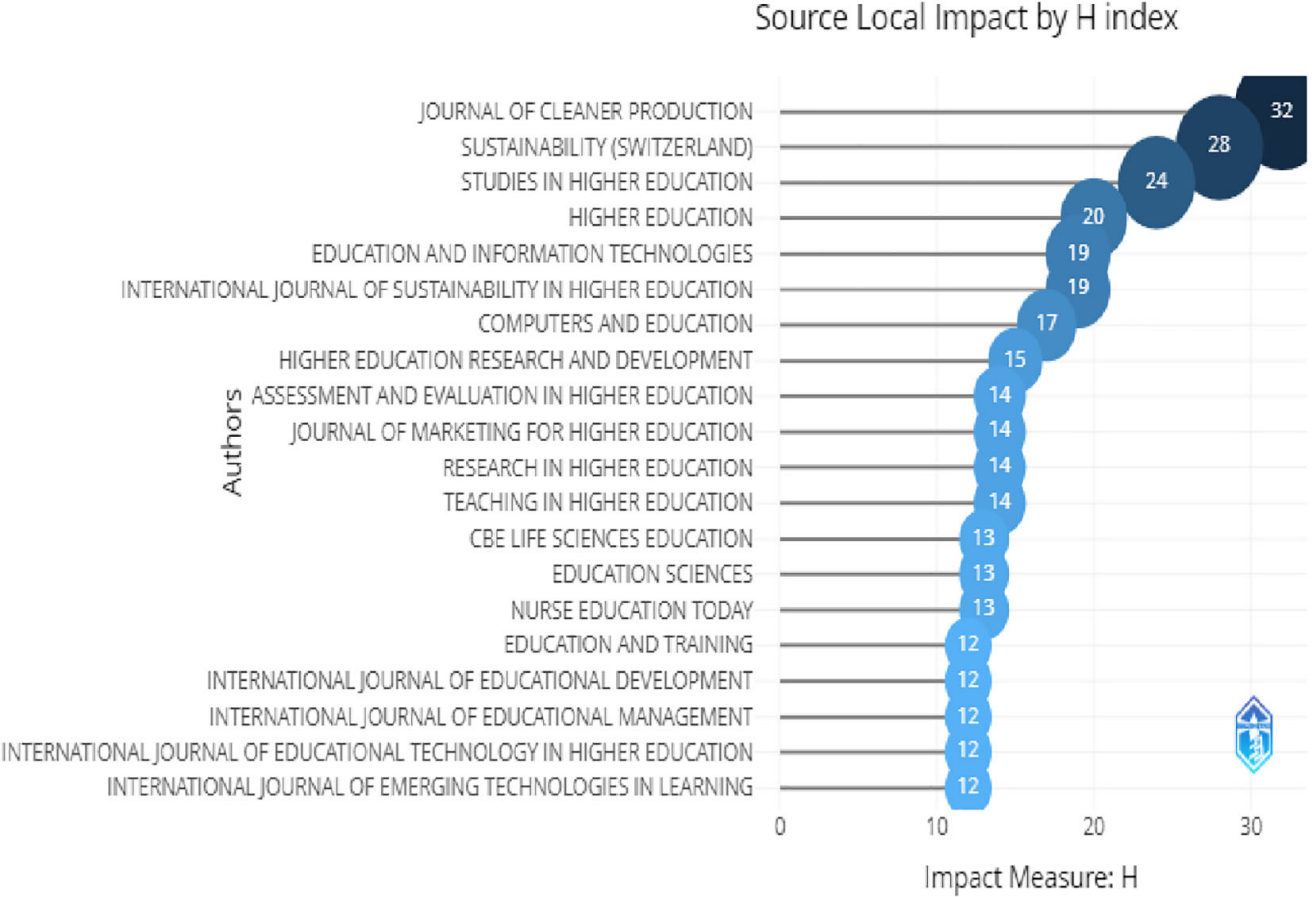
Top 20 impactful sources.

The above Table 3, shows 10 most relevant sources and their impact factor, also in Figure 5, we can see the most relevant sources in which sustainability, studies in higher education, higher education, International Journal of Sustainability in higher education are at the top of the list. Looking at this information we can say that most of the words used here were related to higher education, higher education and technology, advertising, information and online learning.

**Table 3.** Most relevant sources.

Journal name	Impact factor	H-INDEX
Sustainability (Switzerland)	3.889	136
Studies in Higher Education	5.20	120
Higher Education	3	118
International Journal of Sustainability in Higher Education	4.12	72
Education Sciences	3.66	40
Education and Information technologies	3.666	61
IEEE Global Engineering Education Conference Educon	1.57	30
Service Industries Journal	9.40	76
Administrative Sciences	3.38	28
European Journal of Marketing	4.66	146

**Figure 6.**
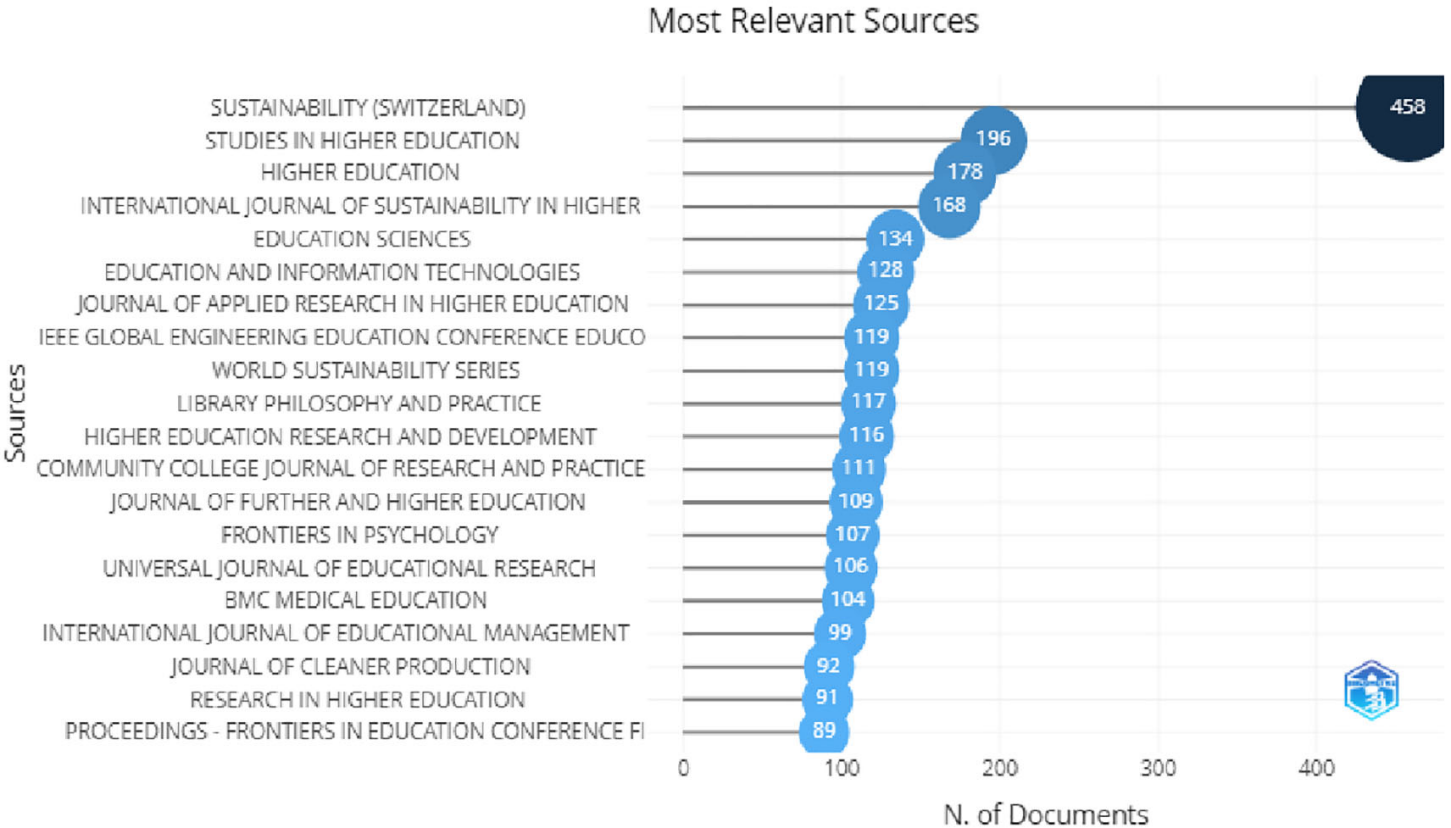
Relevant sources.

Figure 6 shows the effect of these journals. The H-index refers to the most extreme value of “n”. Now, “n” refers to the number of journals which have articles published with the least citations. The H-index indicates the quality of the journal as well as its impact.

*Authors*

**Figure 7.**
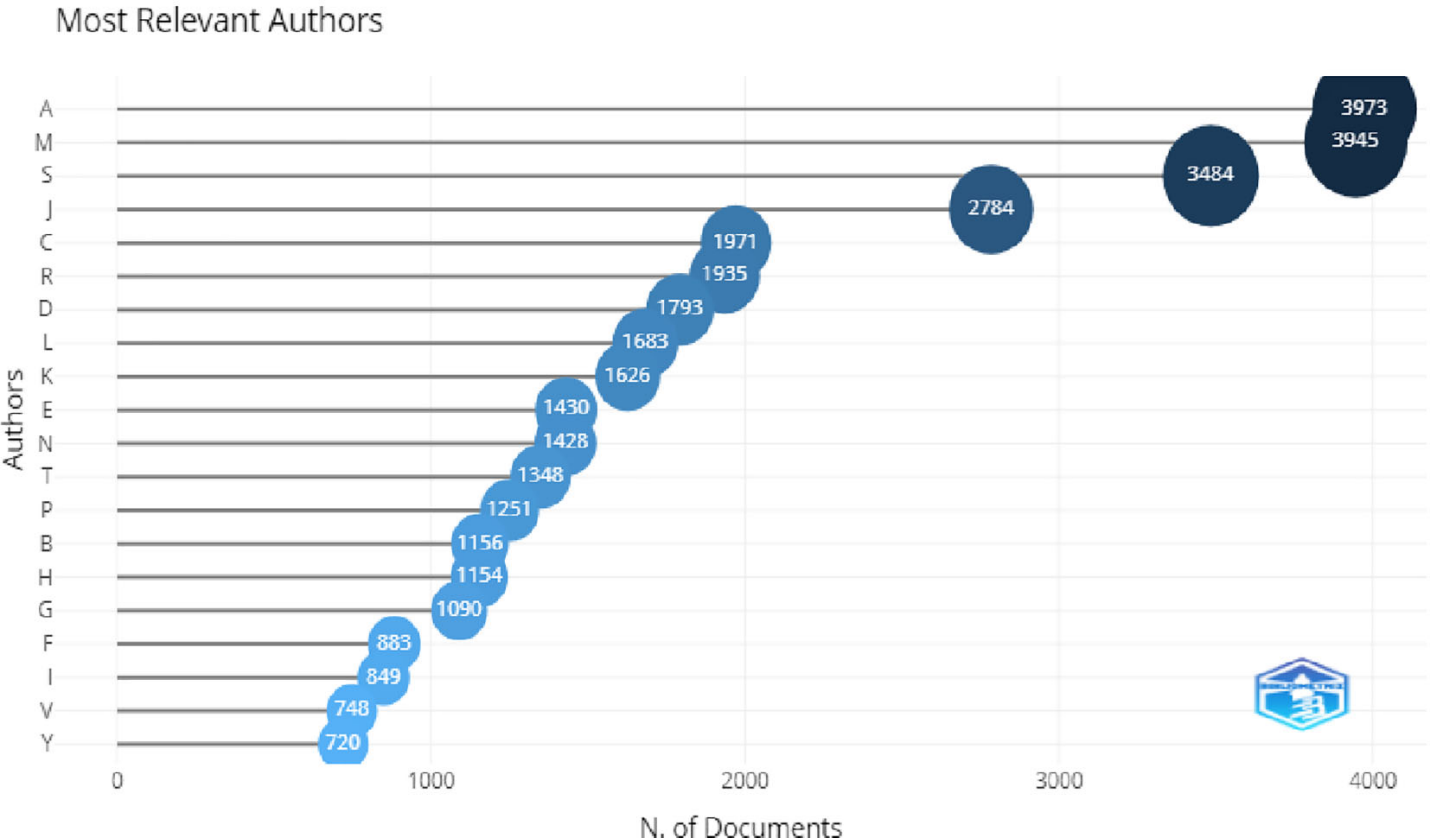
Most relevant authors.

**Figure 8.**
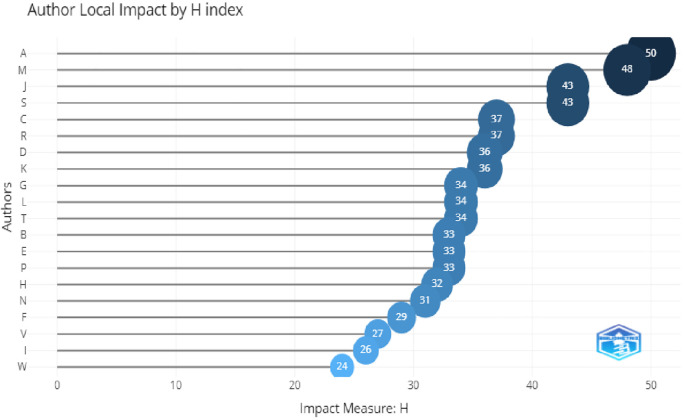
Author impact.

In Figure 7, the top three most relevant authors were A. Aleksander, L. Mishra, and Lou, with the maximum number of publications. The H-index of these authors and citations were also higher, which shows their relevancy. This can be seen in Figure 8.

*Documents*

**Figure 9.**
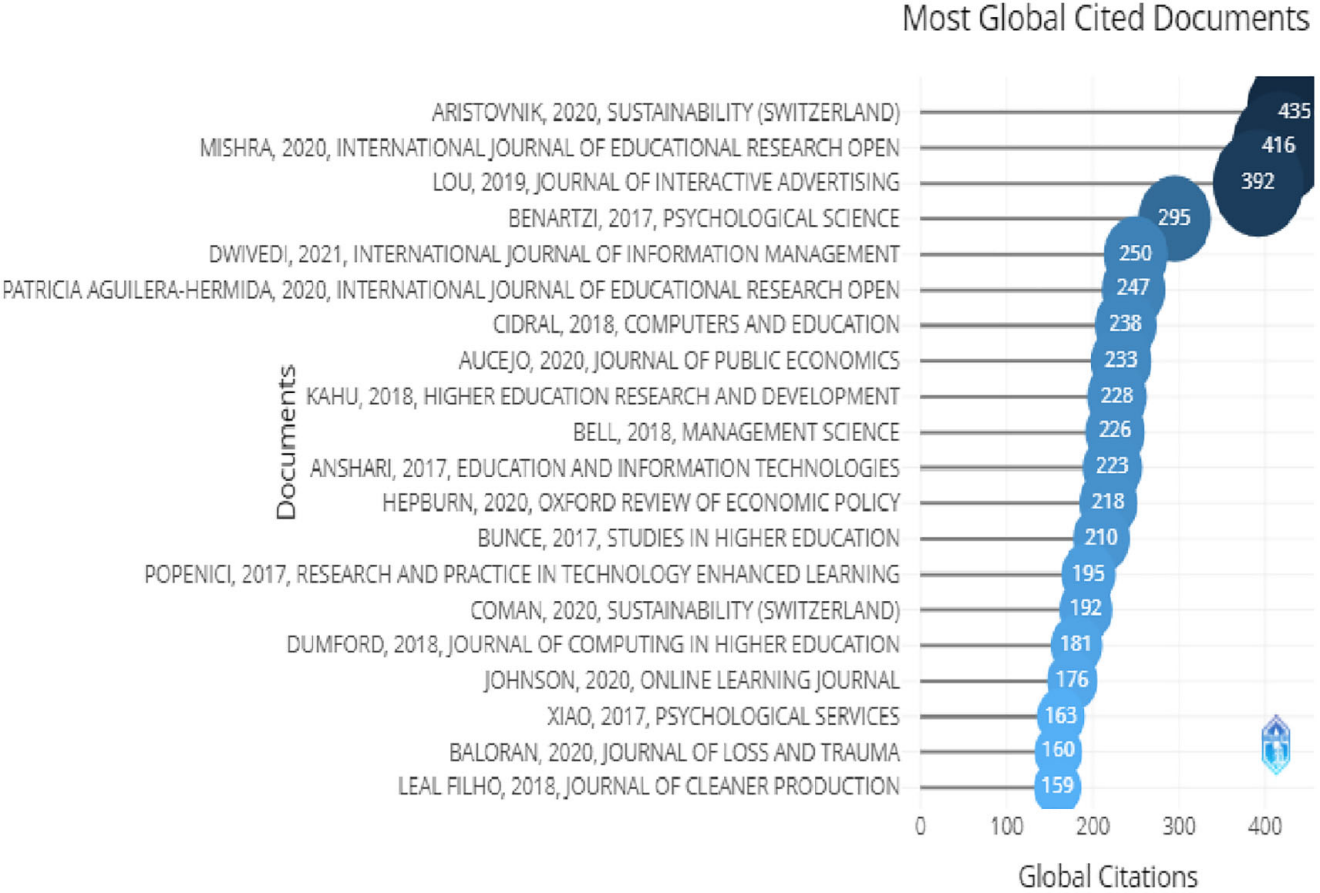
Most globally cited documents.

Figure 9 shows the most cited documents with the author’s name, in this field. There was no article with less than 100 citations and Aristovnik, Mishra and Lou had more than 300 citations. These articles were about advertising, which covered branding and online marketing, higher education, and higher education institutions.

### Defining the appropriate search terms

The main words that were used were “brand awareness”, “higher education institution”, “online marketing strategies”, “online brand awareness and “college enrollment”. Also, to get a better result, words were combined and used for searching. These combinations were 1) online marketing of higher education, 2) online brand awareness, 3) online student enrollment 4) brand awareness and student enrollment. The combination was used to get the appropriate studies. For example, brand awareness and student enrollment yielded studies related to brand awareness effect on enrollment of students, online marketing showed the various online marketing strategies for online brand awareness and enrollment, and so on. During the search we ensured that all the required work in the study was covered. Subjects included were social sciences, business management and psychology.

## Conclusions

Paré *et al.* (2015) stated that a publication is effective only when it has made some advancement on the studies that have been conducted previously and, by identifying the areas where further research can be done. Scholars use bibliometric analysis for a variety of reasons, such as to uncover emerging trends in article and journal performance, collaboration patterns, and research constituents, and to explore the intellectual structure of a specific domain in the existing literature. The results show that from 2017 to 2022 for HEIs, marketing and advertising have become important, and they have been using them. The previously published literature was used for analysis to retrieve the important sources, writers, and records. The Bibliometix R package, which is a valuable tool for bibliometrics was chosen due to its adaptability and ease of use. The Scopus database was used for creating the data set, given its conventional construction and quality sources of the journals. According to the data, papers were published on these topics but had not reached the stage of maturity; however, in the year 2020 there was a sharp increase in the publication, which eventually went down after that year. In 2020, the world was hit by the COVID-19 pandemic and educational institutions were closed for some time, but later the situation forced the institutions to use digital platforms. The COVID-19 pandemic caused countless setbacks around the world, with significant social and financial effects. UNESCO reported that around one billion students had been compelled to remain at home because of the crisis, and the scale and speed with which schools and colleges had been shut was overwhelming ( Giancola and Piromalli, 2020). To face this crisis, even schools and colleges could not stop from transferring their activities to digital platforms ( Turcanu *et al.*, 2020). Most of the publications were from sources like higher education, higher education advertising and technology. This paper, therefore, has given a path for interdisciplinary approaches that can be further explored in the field of higher education and marketing. Further, this paper was an opportunity to examine the pattern of publications by investigating the different authorship, co-authors, collaborations, relevant sources, and citations. The insights of this paper will help education policymakers to devise more creative strategies to increase enrollment. This would give an in-depth understanding of this field. Further, for academic researchers, this paper will open many ways in which one could explore and contribute to the educational industry by diversifying the research in areas where the publications have not reached their maturity level. A combination of keywords was used in this paper: 1) online marketing of higher education, 2) online brand awareness, 3) online student enrollment 4) brand awareness and student enrollment. The bibliometric analysis showed that higher education and marketing are topics which have been explored, resulting in related papers being published, but the effects of online marketing of higher education on students’ enrollment and brand awareness role in higher education and its importance are areas which have not been explored. So, our study gives a path for further research.

### Discussion and Direction for Future Research

The unprecedented challenges that digitalization brought to the world forced higher education institutions also to transform their working style. The study used bibliometric analysis, but only descriptive analysis has been done. The pieces of literature in the study have shown that online marketing and brand awareness are pivotal for higher education institutions to survive and gain a competitive edge and by utilizing Bibliometix R-bundle the study has highlighted the importance of interdisciplinary approaches required in the field. Further, the epidemic that covered the world in the year 2020 (Covid-19), emphasized the profound impact of using online strategies in higher education. Therefore, a literature review has been conducted in the study to provide a panoramic view of the evolving landscape. Bibliometric analysis done in the study by using the Scopus database has traced scholarly work surrounding online marketing and brand awareness in higher education. It was noted that there was a surge in publication in the year 2020, and this was the year when the world was hit by the pandemic. This became the very reason for the education industry to shift its focus on online marketing and brand awareness as there was no escaping from it. The study not only highlights the untapped research avenues but also supports interdisciplinary collaboration in this area of study. The year after 2021 shows that publications in this area are dropping whereas now is the time when more can be explored in this area.

So, this study can be carried out further and can be examined more by using network analysis. Further, the database that has been used for this study is Scopus, whereas other very good databases also can be used like the Web of Science. From this study, we can see that there are not many publications that involve higher education, online marketing brand awareness and student enrollment altogether but in today’s time these are the activities that are helping the colleges in facing the competition. Academic scholars can diversify this research and explore this area more with new technologies like Artificial Intelligence and Generative artificial intelligence. There is a need to explore the contribution that has been made by scholars and practitioners in this field to get deeper theoretical and practical insights.

## Data Availability

Figshare: Online Marketing And Brand Awareness For HEI: A Review And Bibliometric Analysis, https://doi.org/10.6084/m9.figshare.21276603 ( Bohara *et al.*, 2022). This project contains the following underlying data:
• Data file 1. (CSV. File of the bibliometric analysis done using Scopus database) Data are available under the terms of the Creative Commons Zero “No rights reserved” data waiver (CC0 1.0 Public domain dedication).
