# Supplementary material for: Online marketing and brand awareness for HEI: A review and bibliometric analysis
Source: F1000Res. 2024 May 13;12:76. Originally published 2023 Jan 19. [Version 2] doi: 10.12688/f1000research.127026.2 (PMC11809636; doi:10.12688/f1000research.127026.2)
Supplement: Peer review history

# Reviewer report: Reviewer response for version 2

**DOI:** 10.5256/f1000research.164788.r277426

**Published:** 2024-12-24

**Authors:** Arun Vijay Subbarayalu

**Affiliations:** 1 Imam Abdulrahman Bin Faisal University, Dammam, Eastern Province, Saudi Arabia

I have reviewed all the updates in the revised article, and the authors have addressed most of my suggestions and comments. The revised version is much improved, and the manuscript can be considered for indexing in its current form.

Is the work clearly and accurately presented and does it cite the current literature?

Partly

If applicable, is the statistical analysis and its interpretation appropriate?

Partly

Are all the source data underlying the results available to ensure full reproducibility?

Yes

Is the study design appropriate and is the work technically sound?

Partly

Are the conclusions drawn adequately supported by the results?

Partly

Are sufficient details of methods and analysis provided to allow replication by others?

Partly

Reviewer Expertise:

Healthcare Management, Business Administration, Quality Management.

I confirm that I have read this submission and believe that I have an appropriate level of expertise to confirm that it is of an acceptable scientific standard.

# Reviewer report: Reviewer response for version 2

**DOI:** 10.5256/f1000research.164788.r277425

**Published:** 2024-06-04

**Authors:** Sanjib Biswas

**Affiliations:** 1 Decision Sciences, & Operations Management, Calcutta Business School, Kolkata, India

I have seen the revision. I am satisfied.

Is the work clearly and accurately presented and does it cite the current literature?

Partly

If applicable, is the statistical analysis and its interpretation appropriate?

Partly

Are all the source data underlying the results available to ensure full reproducibility?

Yes

Is the study design appropriate and is the work technically sound?

Partly

Are the conclusions drawn adequately supported by the results?

No

Are sufficient details of methods and analysis provided to allow replication by others?

Partly

Reviewer Expertise:

Decision Science, Operations Management

I confirm that I have read this submission and believe that I have an appropriate level of expertise to confirm that it is of an acceptable scientific standard.

# Reviewer report: Reviewer response for version 1

**DOI:** 10.5256/f1000research.139492.r256295

**Published:** 2024-03-29

**Authors:** Arun Vijay Subbarayalu

**Affiliations:** 1 Imam Abdulrahman Bin Faisal University, Dammam, Eastern Province, Saudi Arabia

I would like to express my special thanks to the editors for providing me with the opportunity to review this manuscript. 

 The authors have made a good effort in exploring the concepts of online marketing and brand awareness for higher education institutions (HEI) through a bibliometric approach. I acknowledge the efforts made by the authors in bringing out this study. However, there are still some issues that need to be addressed before it can be accepted for publication in F1000 Research.

 I have provided my comments with regard to each section of the manuscript. Recommendations to be addressed by the authors in the manuscript is given below: 

 Abstract Section:

1. Background: Write the objective of this study clearly to inform the readers what you want to accomplish through this bibliometric study.
2. In the method section, it was mentioned that five years were covered. Nevertheless, the coverage period is six years, from 2017 to 2022.
3. In the results, the author needs to reflect significant findings in the form of data observations retrieved from this study, as the abstract is a concise version (summary) of the manuscript.

Introduction Section

1. It was stated on page 4 that no bibliometric study was done earlier on this topic of study. However, several other studies have been found in the literature on similar lines of research. It would be better to state the research gap concerning the utility of bibliometric analysis focusing on online marking and brand awareness using various databases such as Scopus and WoS. In the introduction, the author has to justify why this study was conducted and what it attempts to fulfill (considering the gap identified by earlier studies).

Methods Section

1. Mention the study design adopted in the methods section.
2. Mention the key terms and Boolean operators used for electronic search criteria within the Scopus database. Also, include the Scopus query used to retrieve the data.
3. Mention the inclusion criteria used to screen documents to be included in the bibliographic analysis within the text under the search strategy.
4. It is essential to mention the date you carried out the search and downloaded the data since it could be replicated by other researchers in the same or other fields.
5. On page 6, under the bibliometric tool, add the software name Biblioshiny in Rstudio.
6. The authors did not mention the Scopus downloaded data type. (For example. CSV or BibTex). Mention it.  Mention it.

Results

1. Write a short note about the retrieved information studied to reflect on the Author, sources, and documents, as mentioned in Figure 2.
2. On page 8, it was mentioned that in Figure 4, the top 20 globally most cited documents can be seen. Sustainability, International Journal of Education, Journal of Advertising, (mismatch). This information does not match the figure-4 provided on page number 8. Correct and update it.
3. Mention the Author's Full name in Figures 7 and 8. It is recommended that a table showing the top 10 authors with their affiliation, frequency (number of documents), percentage (%) of publication concerning the total, and the total citations received by each Author be created.
4. Besides Figure 6, a table showing the top journals published documents related to online marketing and brand awareness for HEI by various authors between 2017 and 2022 would be desirable.
5. Besides Figure 9, it is recommended to create a table showing the top cited documents with their Digital Object Identifier (DOI), total citations received per document, and citations received per year for the clarity of the readers. You can take the top 10 highly cited documents.

Discussion Section.

1. The discussion of findings needs to be elaborated. The authors need to discuss the major findings about the author, sources, and documents, critically appraise, and conclude them.
2. Under the direction of the future research section, consider adding future studies that could focus on country-wise scientific output on online marketing and brand awareness for HEI by various authors, keyword analysis using VOSviewer, and author collaborations on the same research topic.

Is the work clearly and accurately presented and does it cite the current literature?

Partly

If applicable, is the statistical analysis and its interpretation appropriate?

Partly

Are all the source data underlying the results available to ensure full reproducibility?

Yes

Is the study design appropriate and is the work technically sound?

Partly

Are the conclusions drawn adequately supported by the results?

Partly

Are sufficient details of methods and analysis provided to allow replication by others?

Partly

Reviewer Expertise:

Higher Education Quality Management; Healthcare Management.

I confirm that I have read this submission and believe that I have an appropriate level of expertise to confirm that it is of an acceptable scientific standard, however I have significant reservations, as outlined above.

# Response

**Published:** 2024-04-08

**Authors:** Sailaja Bohara

**Affiliations:** Uttaranchal University, India

All the suggestions given have been taken into consideration and all the necessary changes as suggested have been made.

# Reviewer report: Reviewer response for version 1

**DOI:** 10.5256/f1000research.139492.r176356

**Published:** 2023-06-22

**Authors:** Sanjib Biswas

**Affiliations:** 1 Decision Sciences, & Operations Management, Calcutta Business School, Kolkata, India

The topic is interesting. Thank you for providing me an opportunity to go through the article. The authors are requested to address the following concerns:

1. The introduction section is too short to establish the research rationale and the contribution of the paper.
2. The author should use a theoretical framework lilke PRISMA for scientifically describing the research design.
3. Network analysis could have been better.
4. Please extensively elaborate on the discussion and future direction section.
5. Although the authors referred to Scopus and WoS database, the present article needs to address some important articles in the stated area (not published in Scopus but available in google scholar). For instance: Biswas (2020 ^1^).

Is the work clearly and accurately presented and does it cite the current literature?

Partly

If applicable, is the statistical analysis and its interpretation appropriate?

Partly

Are all the source data underlying the results available to ensure full reproducibility?

Yes

Is the study design appropriate and is the work technically sound?

Partly

Are the conclusions drawn adequately supported by the results?

No

Are sufficient details of methods and analysis provided to allow replication by others?

Partly

Reviewer Expertise:

Decision Science, Operations Management

I confirm that I have read this submission and believe that I have an appropriate level of expertise to confirm that it is of an acceptable scientific standard, however I have significant reservations, as outlined above.

## References

[1] : Exploring the Implications of Digital Marketing for Higher Education using Intuitionistic Fuzzy Group Decision Making Approach. *BIMTECH Business Perspective (BSP)* .2020;1-19 Reference source

# Response

**Published:** 2024-04-08

**Authors:** Sailaja Bohara

**Affiliations:** Uttaranchal University, India

The suggestions given has been taken into consideration.
